# In silico antimicrobial resistance profiling of 1,240 *Stenotrophomonas maltophilia* genomes: AMR gene prevalence, temporal evolution, and ST-specific associations

**DOI:** 10.1371/journal.pone.0350669

**Published:** 2026-07-09

**Authors:** Laith B. Alhusseini, Firas Nabeeh Jaafar, Ahmed R. Alsharmani, Ebrahim Kouhsari, Mohammad Sholeh

**Affiliations:** 1 Department of Ecology, College of Science, Kufa University, Kufa, Najaf, Iraq; 2 Microbiology Department, College of Science, Mustansiriyah University, Baghdad, Iraq; 3 Department of Laboratory Sciences, Faculty of Paramedicine, Golestan University of Medical Sciences, Gorgan, Iran; 4 Laboratory Sciences Research Center, Golestan University of Medical Sciences, Gorgan, Iran; 5 Department of Bacteriology, Pasteur Institute of Iran, Tehran, Iran; 6 Student Research Committee, Pasteur Institute of Iran, Tehran, Iran; Laurentian University, CANADA

## Abstract

*Stenotrophomonas maltophilia* is an opportunistic pathogen with clinically important multidrug resistance, yet large-scale genome-based analyses integrating population structure, resistance determinants, and epidemiological metadata remain limited. We analyzed 2,419 publicly available genomes retrieved from NCBI GenBank. After quality control with CheckM, 2,389 assemblies passed filtering criteria, and species identity was further evaluated by average nucleotide identity using FastANI. A final curated dataset of 1,240 confirmed *S. maltophilia* genomes was retained for downstream analyses. Antimicrobial resistance determinants were identified using AMRFinderPlus, sequence types were assigned by multilocus sequence typing, and temporal, geographic, host-associated, and gene co-occurrence analyses were performed. The 1,240 genomes represented isolates collected between 1900 and 2025 from 40 countries. Geographic metadata were available for 1,239 isolates, host source for 1,239 isolates, and collection year for 1,105 isolates. Human-derived isolates predominated, whereas animal-derived isolates were rare and environmental isolates were absent. MLST assigned 876 isolates to 97 sequence types, with ST5, ST4, ST1, ST31, and ST162 as the most prevalent lineages. The resistome comprised 69 unique resistance-associated genes across 12 functional classes and showed a bimodal structure, with a highly conserved intrinsic core and a sparse accessory component. Core genes included *emrA*, *emrB*, *emrC*, *smeF*, *blaL1*, *aac*(6′)-*Iz*, *aph*(6), *aph*(3′)-*IIc*, *ermB*, and *ermC*, most of which occurred at very high prevalence. In contrast, acquired determinants such as *sul2*, *tet*(G), *aadA2*, *blaNDM-1*, *blaGES-1*, and *blaOXA-74* were infrequent. Plasmid replicons were also uncommon, supporting a predominantly chromosomal resistance architecture. Temporal analyses showed that intrinsic genes were present in the earliest available isolates, whereas acquired genes appeared only in recent decades and generally remained rare. Several acquired genes, including *floR*, *aph*(3′′)-*Ib*, *aph*(6)-*Id*, and *aac*(6′)-*Ib4*, declined over time, while no acquired resistance determinant showed a significant increasing trend. Gene presence pattern and co-occurrence analyses identified dominant conserved resistome configurations and a smaller set of variable accessory modules associated with putative mobile genetic elements. Comparative analysis further showed enrichment of intrinsic efflux-associated determinants in clinical isolates, whereas non-clinical isolates carried a broader diversity of acquired aminoglycoside resistance genes. These findings indicate that the global resistome of *S. maltophilia* is dominated by conserved intrinsic, chromosomally encoded resistance determinants, whereas acquired resistance genes remain rare, sporadic, and lineage-associated. This curated genome-scale framework provides a resource for surveillance and for future studies linking resistome evolution, mobile genetic elements, and genotype-phenotype relationships.

## 1. Introduction

*Stenotrophomonas maltophilia* is an emerging multidrug-resistant opportunistic Gram-negative pathogen of significant clinical importance [1,2]. This organism thrives in aquatic habitats and hospital equipment, facilitating nosocomial transmission to immunocompromised patients, those with underlying respiratory disease, and critically ill hospitalized populations [1,3,4]. Prevalence is particularly elevated in cystic fibrosis patients, reaching 16.8% in the United States, where chronic infection is associated with progressive lung function deterioration [5]. Risk factors include malignancy, immunosuppressive therapy, indwelling catheters, and broad-spectrum antibiotic exposure [6,7]. *S. maltophilia* ranks among the top three non-fermentative Gram-negative bacilli in hospitalized patients [8], establishing its importance as a nosocomial pathogen.

The primary challenge posed by *S. maltophilia* is its intrinsic multidrug resistance, mediated by chromosomally encoded determinants rather than acquired genes [9,10]. The organism produces intrinsic L1 and L2 metallo-β-lactamases conferring resistance to carbapenems, rendering standard β-lactam therapy ineffective despite apparent in vitro susceptibility [11]. Resistance to fluoroquinolones, aminoglycosides, and trimethoprim-sulfamethoxazole further restricts therapeutic options [9]. This intrinsic resistance architecture, characterized by constitutive β-lactamase production and multidrug efflux pumps, fundamentally distinguishes *S. maltophilia* from other multidrug-resistant Gram-negative pathogens such as *Pseudomonas aeruginosa* and *Acinetobacter baumannii*, which rely more heavily on acquired mechanisms [12,13]. The chromosomal localization of resistance genes makes them difficult to eliminate through selective pressures [13], and *S. maltophilia* can acquire additional resistance determinants, further limiting therapeutic options [14,15]. Current Infectious Diseases Society of America (IDSA) guidelines advise against ceftazidime monotherapy despite apparent susceptibility, reflecting the clinical unreliability of standard susceptibility testing [11]. Recent therapeutic advances may provide solutions to this impasse. Ceftazidime-avibactam, a combination of the third-generation cephalosporin ceftazidime with the novel non-β-lactam β-lactamase inhibitor avibactam, demonstrates in vitro activity against many *S. maltophilia* isolates carrying *blaL1* [13]. Additionally, cefiderocol, a fourth-generation siderophore-conjugated cephalosporin designed to enhance iron uptake-mediated penetration, shows promise against multidrug-resistant Gram-negative organisms including *S. maltophilia* [16,17]. However, clinical experience with these agents in *S. maltophilia* infections remains limited, and optimal dosing, pharmacodynamic targets, and clinical efficacy require definition through prospective clinical trials. Thus, comprehensive characterization of underlying resistance mechanisms remains urgent for optimizing treatment strategies, predicting resistance phenotypes from genomic data, and developing future therapeutic approaches.

Despite clinical significance, *S. maltophilia* remains substantially undercharacterized genomically compared to other Gram-negative organisms [18,19]. Critical knowledge gaps include: (1) poorly defined global distribution and phylogeographic patterns of major sequence types (STs) [20] (2) uncharacterized temporal evolution of resistance gene prevalence in clinical populations; (3) inadequately documented geographic and clonal associations of specific resistance determinants [21]; (4) incompletely understood mechanisms of horizontal gene transfer and resistance acquisition [22]; and (5) unestablished relationships between genomic features, resistance phenotypes, and clinical outcomes [23]. Unlike well-characterized pathogens such as *Salmonella enterica* and *Pseudomonas aeruginosa*, which have standardized genomic frameworks enabling international outbreak tracking [24,25], *S. maltophilia* lacks comparable epidemiological infrastructure. The narrow core genome comprising only 11.6% of the pangenome reflects substantial clonal diversity and strain-specific variation [18], yet this heterogeneity has not been systematically mapped to clinical phenotypes. Consequently, clinicians cannot reliably predict susceptibility from genomic data, and infection control personnel lack standardized molecular tools for outbreak detection. Establishing WGS-based surveillance networks and characterizing resistance gene prevalence and clonal relationships are essential priorities.

In silico whole-genome analysis and comparative genomics enable unprecedented characterization of pathogen population structure, evolutionary dynamics, and resistance mechanisms [26,27]. WGS facilitates identification of antimicrobial resistance genes through tools such as AMRFinderPlus, enabling rapid detection of known and novel determinants [28,29]. MLST and phylogenomic reconstruction using core genome SNPs provide refined clonal relationships superior to traditional methods [30]. Integration of genomic data with epidemiological metadata enables temporal trend analysis revealing how selective pressures shape resistance gene distribution [31]. Phylogeographic analysis maps spatial dissemination patterns and transmission chains impractical through conventional methods [32]. Machine learning classifiers trained on whole-genome sequence data predict antimicrobial resistance profiles with substantial accuracy [33], while comparative genomics identifies genetic elements associated with resistance phenotypes [34,35]. The scalability of in silico analysis handles geographically diverse datasets prohibitive through laboratory methods [26,27], enabling improved epidemiological resolution for outbreak detection and susceptibility prediction [36,37]. These advantages establish in silico genomics as essential infrastructure for bacterial epidemiology and resistance surveillance.

This study addresses critical knowledge gaps by comprehensively characterizing high-quality whole-genome assemblies from 57 countries, representing the largest curated *S. maltophilia* collection to date [18,19]. Objectives include: (1) characterizing global population structure through MLST analysis; (2) quantifying prevalence of 92 antimicrobial resistance genes and identifying ST-specific profiles [28]; (3) analyzing temporal trends in resistance gene prevalence to infer evolutionary responses to changing antibiotic selective pressures; and (4) integrating genomic data with epidemiological metadata to elucidate transmission dynamics [38–40]. This approach leverages established methodologies from multidrug-resistant pathogen surveillance [41–43], enabling standardized comparative analysis and international integration. By examining relationships between genome size, gene content, resistance prevalence, and epidemiological characteristics, this study establishes the genomic basis of *S. maltophilia* population structure and phenotypes [44,45]. Temporal analysis provides unique evolutionary perspective on how antibiotic selective pressures have shaped resistance gene prevalence and clonal composition [42], while integration with MLST frameworks establishes infrastructure for future surveillance and stewardship [39]. The curated genomic repository with integrated metadata enables future epidemiological investigations and mechanistic studies of bacterial adaptation [40,46], comparable to successful surveillance frameworks for other pathogens [47,48]. This study transforms *S. maltophilia* from undercharacterized pathogen into a well-defined genomic epidemiological entity, enabling evidence-based clinical and public health responses to this multidrug-resistant threat.

## 2. Methods

### 2.1 Genome retrieval, metadata curation, and species confirmation

Whole-genome assemblies annotated as *Stenotrophomonas maltophilia* were retrieved from the NCBI Genome/Assembly database in FASTA format together with associated metadata from linked NCBI Assembly and BioSample records. Retrieved metadata included accession number, strain or isolate name, assembly status, collection year, country or geographic origin, and available source information. All accession numbers and curated isolate-level metadata used in this study were provided in the supplementary file to ensure full traceability and reproducibility.

Initial genome quality assessment was performed using CheckM v1.2.3 under the lineage-specific workflow. Assemblies passing predefined quality thresholds for completeness and contamination were retained for downstream processing. Because members of the *S. maltophilia* complex can be misannotated in public repositories, species identity was further confirmed using FastANI. Average nucleotide identity values were calculated against an appropriate *S. maltophilia* reference genome, and only genomes meeting the species-level ANI threshold were retained. This ANI confirmation step was used to exclude potentially misclassified non-*S. maltophilia* assemblies before comparative analyses.

Metadata were manually curated and standardized after quality and taxonomic filtering. Country names were harmonized, collection years were checked for formatting consistency and biologically implausible values, and host/source fields were normalized into broader analytical categories. Isolates lacking a valid collection year were excluded only from year-based analyses and retained for non-temporal analyses when other required metadata were available.

### 2.2 Assembly feature extraction and genome characteristics

Assembly characteristics for all retained genomes were extracted directly from NCBI Assembly records rather than being re-annotated de novo. Variables collected included assembly level, genome size, GC content, contig count, scaffold count where available, contig N50, scaffold N50, and NCBI-reported gene annotation summaries such as total genes, coding sequences, and pseudogenes. Because these values originated from NCBI database records, they reflect the deposited assembly and annotation status of each genome at the time of retrieval.

Descriptive statistics were calculated across the curated genome set to summarize assembly heterogeneity and structural variation. These metrics were also used in quality-aware interpretation of downstream comparisons, particularly where fragmented assemblies could influence apparent gene counts or locus recovery.

### 2.3 Antimicrobial resistance gene detection

Antimicrobial resistance determinants were identified using AMRFinderPlus with default curated-reference matching logic appropriate for bacterial genome screening. Resistance genes were recorded as present when sequence similarity and coverage criteria satisfied AMRFinderPlus reporting thresholds. The resulting output was manually curated to consolidate gene names, remove formatting inconsistencies, and classify genes into biologically relevant resistance groups, including intrinsic efflux-associated determinants, beta-lactamases, aminoglycoside resistance genes, sulfonamide/trimethoprim resistance genes, tetracycline resistance genes, phenicol resistance genes, quinolone-associated determinants, and rare acquired beta-lactamases.

The final AMR matrix was converted into a binary presence/absence table for isolate-level and lineage-level analyses. Gene prevalence was calculated as the proportion of genomes carrying each determinant. We interpreted near-universal determinants, such as those representing known intrinsic resistance in *S. maltophilia*, separately from rare accessory or putatively acquired genes.

### 2.4 Sequence typing and lineage assignment

Multilocus sequence typing was performed using the established *S. maltophilia* MLST scheme and publicly available allele definitions. Assemblies were screened for the seven housekeeping loci of the scheme, and sequence types were assigned when complete and unambiguous allele combinations were detected. Isolates with incomplete loci, ambiguous matches, or assembly fragmentation affecting allele recovery were classified as untypeable in the MLST-based analysis.

Sequence type frequency distributions were summarized across the dataset, and major sequence types were used in comparative resistome analyses. Because the dataset included assemblies of variable contiguity, we interpreted MLST failure primarily as a technical limitation of incomplete genome representation rather than as biological absence of housekeeping loci.

### 2.5 Comparative resistome profiling and lineage-level analysis

Comparative resistome analysis was based on the isolate-by-gene binary matrix generated from AMRFinderPlus output. For major sequence types and other relevant isolate groupings, gene prevalence was aggregated and compared to identify conserved, variable, and rare resistance determinants. Hierarchical clustering of gene presence/absence profiles was used to assess similarity among sequence types and to identify shared resistome architectures.

This study did not use Prokka or Roary, and no pan-genome reconstruction was performed. Accordingly, comparative analyses were restricted to curated resistance gene distributions and associated metadata rather than whole-pan-genome inference. This analytical scope was chosen to address resistome prevalence, temporal behavior, and sequence type–associated patterns in a reproducible manner using uniformly retrievable public genomes.

### 2.6 Mobile genetic element–related resistance context

To explore the possible mobility of accessory resistance determinants, genes with known or suspected associations with mobile genetic elements were examined separately from the intrinsic resistome backbone. This analysis focused on rare acquired genes, co-occurring accessory profiles, and genes previously linked in the literature to integrons, transposon-associated regions, plasmid-related structures, or other mobile contexts. Because long-read assemblies and complete neighborhood reconstruction were not consistently available for all genomes, the present study did not attempt full structural resolution of mobile genetic elements. Instead, the analysis was used to distinguish stable chromosomal intrinsic determinants from resistance genes showing distribution patterns suggestive of horizontal acquisition.

Where relevant, plasmid-associated replicon signals and accessory AMR clustering patterns were interpreted as indirect evidence for mobility, with clear acknowledgment that definitive structural confirmation would require dedicated mobile-element analysis using high-contiguity assemblies.

### 2.7 Temporal analysis of resistance gene prevalence

Temporal analyses were restricted to isolates with valid collection-year metadata. Annual prevalence of individual resistance genes was calculated from the curated binary matrix. Because public-genome deposition is highly uneven across years, two complementary regression approaches were used: unweighted linear regression and weighted regression incorporating annual isolate counts. In addition, years represented by fewer than two isolates were removed from regression modeling to reduce instability caused by extremely sparse sampling.

Slope estimates, direction of change, and statistical significance were evaluated for genes with sufficient annual representation. Temporal findings were interpreted cautiously and considered descriptive of the available genomic archive rather than definitive reconstruction of long-term evolutionary dynamics. This approach was specifically adopted to improve robustness and reduce the influence of isolated historical records or low-sample-year artifacts.

### 2.8 Correlation analysis and metadata-linked comparisons

Correlation analyses were performed to assess relationships among genome structural variables, temporal metadata, and resistance gene distributions. Depending on variable type and distribution, appropriate correlation statistics and group comparisons were applied to examine associations between genome size, gene count, assembly fragmentation, collection year, and resistance determinants. Comparative analyses were also conducted between metadata-defined groups, including clinical versus non-clinical sources where sufficient sample numbers were available.

Because metadata completeness varied across isolates, each comparison was performed on the subset of genomes with valid information for the variables being tested. The denominator used for each analysis therefore differed according to metadata availability, and this was considered explicitly during interpretation.

### 2.9 Co-occurrence analysis and resistance architecture

Gene co-occurrence analysis was performed using the binary AMR matrix to identify recurrent combinations of resistance determinants across isolates. Pairwise co-detection patterns were examined to distinguish tightly conserved intrinsic modules from sporadic accessory combinations. These patterns were used to infer resistome organization, including a core intrinsic resistance backbone and a smaller accessory component with non-random distribution across certain lineages or source groups.

Because the study was based primarily on assembled public genomes rather than complete chromosomal reconstructions, co-occurrence was interpreted as a population-level association measure and not as definitive proof of direct physical linkage. Nevertheless, recurrent co-occurrence of specific accessory genes was used as supporting evidence for shared dissemination routes or common mobile-element association.

### 2.10 Visualization, reproducibility, and data availability

All statistical analyses, figure generation, and data processing were performed using reproducible scripted workflows in R. Visualizations included prevalence summaries, temporal trend plots, geographic distributions, sequence type distributions, heatmaps of gene presence/absence, and co-occurrence-based summaries.

All isolate accession numbers, curated metadata, and analysis-derived summary tables used in this study were made available in the S1 File. These files contain the underlying dataset required to reproduce the descriptive and comparative analyses presented here.

## 3. Results

### 3.1 Dataset composition, quality assessment, and species confirmation

A total of 2,419 *Stenotrophomonas maltophilia* genome assemblies were retrieved from NCBI GenBank and subjected to rigorous quality control. Initial screening using CheckM (completeness ≥95%, contamination ≤5%) excluded 30 assemblies, yielding 2,389 high-quality genomes. To confirm species identity and eliminate potential misclassifications, all retained genomes were subjected to average nucleotide identity (ANI) analysis using FastANI with a stringent threshold of >95% identity against the *S. maltophilia* reference genome. After confirmation with FastANI, **1,240 isolates were retained** for comprehensive downstream analyses. Among these, metadata completeness was high: geographic origin was available for 1,239 isolates (99.9%), host source for 1,239 isolates (99.9%), and collection date for 1,105 isolates (89.1%). This curated dataset represents one of the largest genomic collections of *S. maltophilia* subjected to integrated genomic and antimicrobial resistance profiling to date.

**Fig 1:**
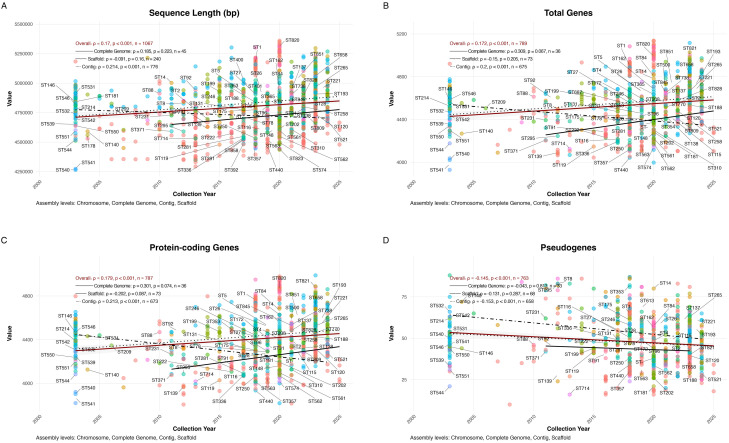
Genomic characteristics and assembly quality of *Stenotrophomonas maltophilia* isolates. Scatter plots showing temporal trends (2000–2025) in genome size (A), total gene count (B), protein-coding genes (C), and pseudogenes (D) across assembly levels (Chromosome, Complete Genome, Contig, Scaffold). **(A)** Genome size ranges 4.25–5.25 Mbp (mean ~4.75 Mbp); overall ρ = 0.17, p < 0.001, n = 1067. Complete genomes show positive correlation (ρ = 0.223, p < 0.001, n = 45); contigs show negative trend (ρ = −0.214, p < 0.001, n = 776). **(B)** Total genes range 4000–5200 (mean ~4400); overall ρ = 0.172, p < 0.001, n = 780, reflecting improved annotation pipelines. **(C)** Protein-coding genes range 3900–4900 (mean ~4300); overall ρ = 0.179, p < 0.001, n = 787. **(D)** Pseudogenes range 10–80 (mean ~40–50); negative correlation (ρ = −0.145, p < 0.001, n = 763) suggests improved assembly quality or annotation stringency in recent years. High variability indicates ST-specific genomic decay or annotation artifacts. Representative STs labeled across panels.

Assembly quality metrics revealed considerable heterogeneity in sequencing depth and completeness (Fig 1). Among the 1,240 analyzed genomes, the majority were contig-level assemblies (n = 883, 71.2%), followed by scaffold-level assemblies (n = 290, 23.4%), complete genomes (n = 56, 4.5%), and chromosome-level assemblies (n = 11, 0.9%). Mean genome size was 4,776,766 bp (SD ± 301,467.8 bp; range 1,774,728–6,641,610 bp), consistent with the known genomic architecture of *S. maltophilia*. Temporal analysis of genome size (2000–2025) revealed an overall positive correlation (ρ = 0.17, p < 0.001, n = 1,067), with complete genomes showing stronger correlation (ρ = 0.223, p < 0.001, n = 45) while contig-level assemblies exhibited a negative trend (ρ = −0.214, p < 0.001, n = 776), likely reflecting assembly fragmentation artifacts. GC content was remarkably uniform across isolates, with a mean of 66.39% (range 65.5%–67%), reflecting the species’ characteristic high-GC genome. On average, genomes harbored 4,530.9 annotated genes, including 4,398.45 protein-coding genes and 56.81 pseudogenes. Total gene count showed temporal increase (ρ = 0.172, p < 0.001, n = 780), ranging from 4,000–5,200 genes (mean ~4,400), reflecting improved annotation pipelines over time. Protein-coding genes exhibited similar trends (ρ = 0.179, p < 0.001, n = 787), ranging from 3,900–4,900 genes (mean ~4,300). Notably, pseudogene counts showed a negative temporal correlation (ρ = −0.145, p < 0.001, n = 763), declining from 10–80 genes (mean ~40–50) in early assemblies to lower counts in recent years, suggesting improved assembly quality or annotation stringency. Assembly contiguity varied widely: mean contig N50 was 312,894 bp (range 1,828–5,059,140 bp), and mean scaffold N50 was 383,450.1 bp (range 2,125–5,062,159 bp). While the majority of assemblies were fragmented, the inclusion of 56 complete genomes provided high-resolution reference points for synteny and gene-context analyses. Detailed assembly statistics for all isolates are provided in S1 File.

### 3.2 Geographic distribution and surveillance bias

**Fig 2:**
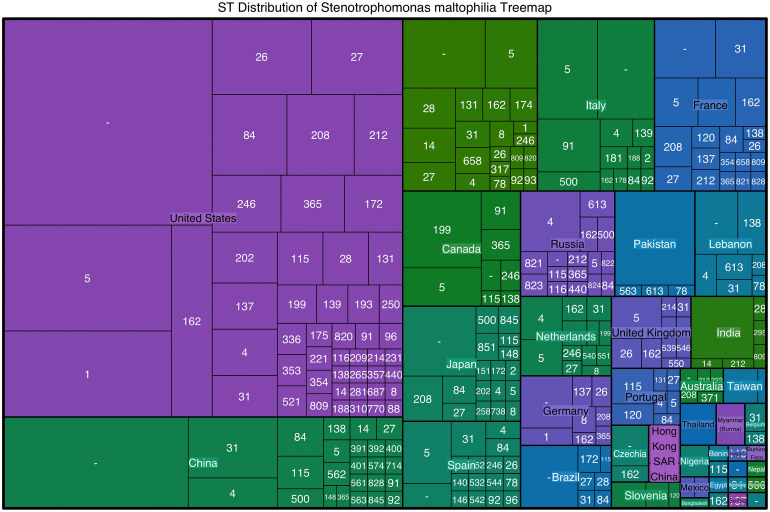
Global distribution and temporal dynamics of *Stenotrophomonas maltophilia* sequence types. **(A)** Treemap depicting geographic and ST distribution across 777 genomes. Rectangle size reflects isolate count; color indicates country. USA dominates with ST246, ST365, ST172, ST84, ST208, ST212. Other major contributors: Canada (ST199, ST365, ST91), China (ST31, ST4, ST500, ST115), Italy (ST500, ST91, ST181), Pakistan (ST613). Cosmopolitan STs (ST4, ST31, ST84) coexist with region-specific lineages. **(B)** Stacked bar chart (1990–2024) showing ST proportional representation (upper panel) and absolute isolate counts (lower panel). Sequencing activity peaks 2018–2020 (>80–100 isolates/year). Early years (1990–2010) show limited diversity; recent years demonstrate marked ST diversification. Labels indicate STs with ≥5% annual prevalence.

Geographic metadata were available for all 1,240 isolates, representing 40 countries across six continents (Fig 2A). However, the dataset exhibited pronounced geographic skew, reflecting disparities in genomic surveillance infrastructure rather than true epidemiological prevalence. The **United States contributed 528 isolates (42.58%)**, representing nearly half of the global collection and dominating with multiple sequence types including ST246, ST365, ST172, ST84, ST208, and ST212. This was followed by China (n = 121, 9.76%), with notable representation of ST31, ST4, ST500, and ST115; Italy (n = 67, 5.40%), harboring ST500, ST91, and ST181; France (n = 64, 5.16%); Canada (n = 45, 3.63%), with ST199, ST365, and ST91; Japan (n = 45, 3.63%); Spain (n = 34, 2.74%); Russia (n = 33, 2.66%); Pakistan (n = 28, 2.26%), predominantly ST613; Lebanon (n = 25, 2.02%); the Netherlands (n = 24, 1.94%); Germany (n = 21, 1.69%); Brazil and the United Kingdom (n = 19 each, 1.53%); and India (n = 18, 1.45%). The remaining 27 countries each contributed fewer than 18 isolates, with many represented by single-digit counts. Notably, 77 isolates (6.21%) lacked country-level annotation, suggesting incomplete metadata curation in public repositories. The treemap visualization revealed that cosmopolitan STs (ST4, ST31, ST84) coexist with region-specific lineages, indicating both global dissemination of high-risk clones and localized endemic transmission. This geographic imbalance underscores the need for expanded surveillance efforts in underrepresented regions, particularly in Africa, South America, and Southeast Asia, where *S. maltophilia* is an emerging nosocomial pathogen but genomic data remain sparse.

### 3.3 Temporal distribution and the genomic surveillance expansion

**Fig 3:**
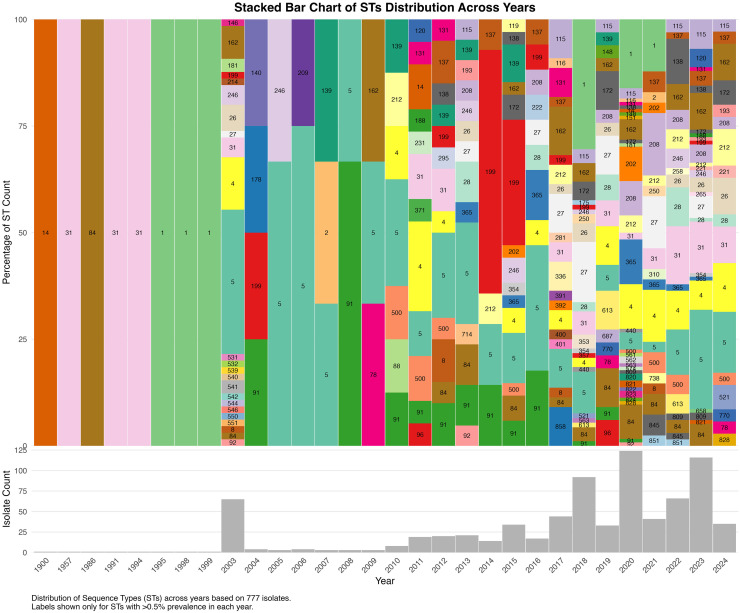
Temporal evolution of *Stenotrophomonas maltophilia* sequence types (1980–2024). Dual-panel visualization of 777 isolates. **(Upper)** Stacked bar chart showing proportional ST representation; labels for STs ≥ 0.5% annual prevalence. **(Lower)** Histogram of isolates sequenced per year. **Key patterns:** (1) Historical dominance (1980–2000): limited diversity (ST14, ST31, ST84); (2) Emergence of high-risk clones (2000–2010): ST199, ST500 persist through 2024; (3) Clonal diversification (2010–2024): > 30 STs circulate annually, including ST365, ST246, ST212, ST162, ST137, ST115, ST208, ST172, ST139, ST138; (4) Surveillance intensification: sequencing increases from ~5 isolates/year (pre-2015) to >100/year (2019–2023), peaking at ~120 (2021); (5) Clonal replacement: transient prevalence patterns (e.g., ST246 peaks 2011–2012) suggest competitive exclusion or differential selection. Transition from monoclonal dominance to polyclonal endemicity mirrors patterns in *Pseudomonas aeruginosa* and *Acinetobacter baumannii*. STs assigned via MLST; data span clinical, environmental, and veterinary sources.

Temporal metadata were available for 1,106 isolates (89.2%), spanning an extraordinary 125-year period from 1900 to 2025 (Figs 2B and 3). However, the temporal distribution was highly uneven, with the vast majority of genomes originating from the past two decades. The earliest isolates were exceedingly rare: the 1900s contributed only 2 isolates (0.16%), the 1950s and 1980s each contributed 1 isolate (0.08%), and the 1990s contributed 7 isolates (0.56%). Historical dominance (1980–2000) was characterized by limited ST diversity, with ST14, ST31, and ST84 representing the primary circulating lineages. Genome availability increased modestly in the 2000s (n = 104, 8.39%), marking the emergence of high-risk clones such as ST199 and ST500, which have persisted through 2024. The dataset surged dramatically in the 2010s (n = 463, 37.34%) and 2020s (n = 528, 42.58%), reflecting exponential growth in whole-genome sequencing capacity. Peak collection years were 2023 (n = 160), 2020 (n = 150), 2018 (n = 128), and 2022 (n = 104), collectively accounting for 35.6% of the entire dataset. Sequencing activity intensified from ~5 isolates/year (pre-2015) to >100 isolates/year (2019–2023), peaking at ~120 isolates in 2021. The year 2023 alone contributed 160 isolates from 8 countries and 27 sequence types, representing the most intensive single-year sampling effort. The period from 2010–2024 demonstrated marked clonal diversification, with >30 STs circulating annually, including ST365, ST246, ST212, ST162, ST137, ST115, ST208, ST172, ST139, and ST138. Transient prevalence patterns, such as the peak of ST246 in 2011–2012, suggest clonal replacement dynamics driven by competitive exclusion or differential selection pressures. This transition from monoclonal dominance to polyclonal endemicity mirrors evolutionary patterns observed in other opportunistic pathogens such as *Pseudomonas aeruginosa* and *Acinetobacter baumannii*. The inclusion of 4 isolates from 2025 indicates ongoing real-time genomic surveillance. Importantly, the scarcity of pre-2000 genomes limits our ability to reconstruct the deep evolutionary history of antimicrobial resistance in this species, as most resistance gene emergence events likely predate the genomic era.

### 3.4 Host source distribution and the human-centric bias

Host metadata were available for 902 isolates (72.7%), while 338 isolates (27.3%) lacked host annotation. Among annotated isolates, the dataset was overwhelmingly human-derived: **871 isolates (70.24%) originated from human sources**, while only **31 isolates (2.5%) were from animal sources**. This 28-fold disparity reflects the clinical focus of *S. maltophilia* genomic surveillance, as the species is primarily recognized as an opportunistic human pathogen associated with healthcare-associated infections, particularly in immunocompromised patients, cystic fibrosis patients, and intensive care unit settings. The paucity of animal-derived isolates (n = 31) limits inference regarding zoonotic reservoirs, environmental niches, or the role of animal agriculture in resistance gene dissemination. Notably, the animal isolates were distributed across multiple host species and geographic regions, suggesting sporadic rather than systematic veterinary surveillance. The absence of environmental isolates (e.g., from soil, water, or plant sources) is a significant limitation, as *S. maltophilia* is ubiquitous in natural environments and environmental reservoirs may serve as ancestral sources of resistance genes. Future studies should prioritize One Health sampling strategies to capture the full ecological breadth of *S. maltophilia* populations.

### 3.5 Multilocus sequence typing and clonal population structure

MLST analysis successfully typed 876 isolates (70.6%), identifying **97 unique sequence types (STs)**. The remaining 364 isolates (29.4%) could not be typed, likely due to incomplete or fragmented assemblies lacking one or more MLST loci. The ST distribution was highly uneven, consistent with a clonal population structure dominated by a small number of globally disseminated lineages. The five most prevalent STs were **ST5 (n = 135, 15.4% of typed isolates)**, **ST4 (n = 62, 7.1%)**, **ST1 (n = 57, 6.5%)**, **ST31 (n = 53, 6.0%)**, and **ST162 (n = 46, 5.3%)**. These five STs collectively accounted for 40.3% of all typed isolates, indicating strong clonal expansion. The top 10 STs (ST5, ST4, ST1, ST31, ST162, ST27, ST84, ST26, ST208, ST199) represented 52.3% of typed isolates, while the remaining 87 STs were comparatively rare. Notably, 58 STs were represented by fewer than 5 isolates each, and 23 STs were singletons, suggesting a long-tail distribution of sporadic or geographically restricted lineages. ST5, the dominant clone, was detected in 10.89% of the entire dataset and was distributed across multiple countries and years, consistent with its status as a globally disseminated high-risk clone. ST4 and ST1, the second and third most common STs, exhibited similar cosmopolitan distributions. In contrast, many rare STs were geographically restricted, suggesting localized transmission or recent emergence. The high ST diversity (97 STs from 876 isolates) indicates substantial genomic heterogeneity within *S. maltophilia*, despite the dominance of a few clones. This pattern is consistent with a mixed epidemic structure, in which globally successful clones coexist with diverse endemic lineages.

### 3.6 Antimicrobial resistance gene landscape: A bimodal resistome

**Fig 4:**
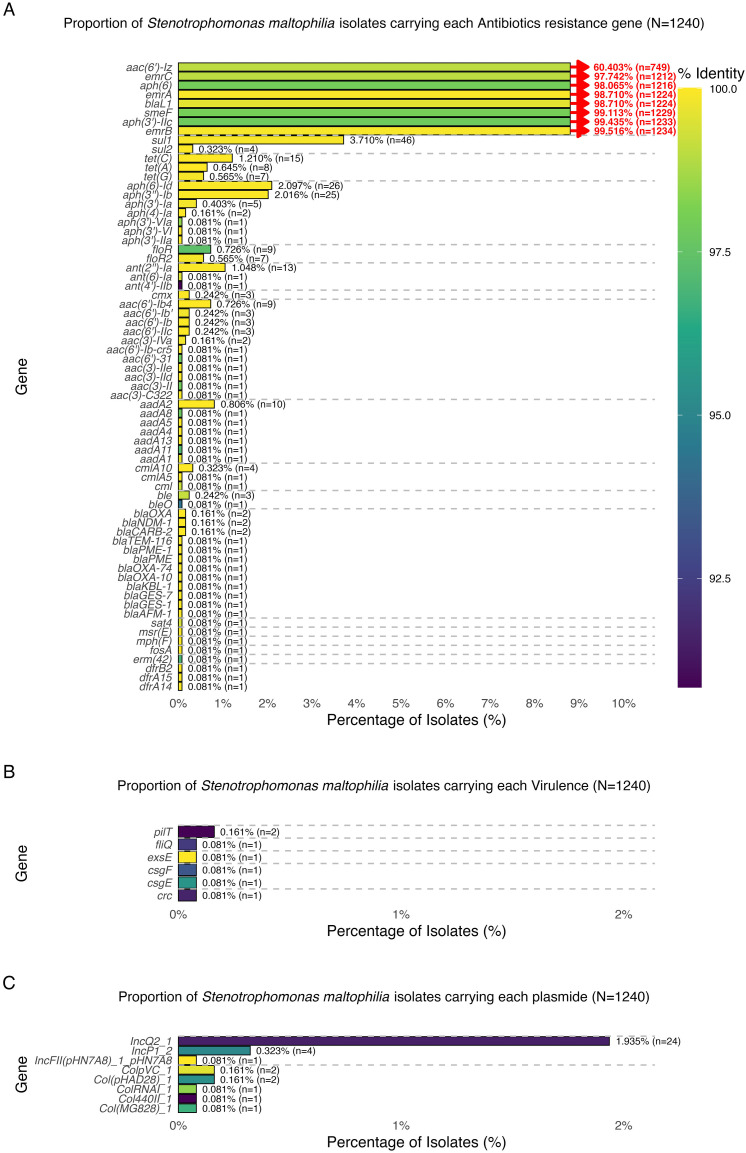
Prevalence of antimicrobial resistance, virulence, and plasmid determinants. **(A)** Horizontal bar chart showing resistance gene prevalence in 1240 isolates; color intensity indicates sequence identity (92.5–100%). Core resistome (>97%): *aac(6’)-Iz* (60.4%), *ermC* (97.7%), *aph(6)* (98.1%), *emrA* (98.1%), *blaL1* (98.7%), *smeF* (99.1%), *aph(3’)-IIc* (99.4%), *ermB* (99.5%), representing intrinsic resistance. Intermediate prevalence (2–10%): *sul2* (3.7%), *aadA2* (0.8%), *tet(G)* (1.1%), indicating acquired resistance. Low-prevalence (<1%): carbapenemases (*blaNDM-1*, *blaOXA-74*, *blaOXA-10*), ESBLs (*blaGES-1*, *blaGES-7*), suggesting sporadic horizontal gene transfer. **(B)** Virulence genes show minimal prevalence: *pilT* (0.16%), *fliQ* (0.08%), *exsE* (0.08%), *csgF* (0.08%), *csgE* (0.08%), *crc* (0.08%). **(C)** Plasmid replicons: IncQ2_1 dominates (1.9%), followed by IncP1_2 (0.3%). Low overall prevalence (<2%) suggests predominantly chromosomal resistance.

AMRFinderPlus identified 69 unique resistance-associated genes across the 1,240 isolates, spanning 12 functional classes (Fig 4A). The resistome exhibited a striking bimodal distribution, with a small number of highly prevalent core genes and a long tail of rare acquired determinants. At the class level, aminoglycoside resistance genes were universally detected (n = 1,240, 100%), followed by efflux pumps (n = 1,236, 99.7%), quinolone resistance determinants (n = 1,229, 99.1%), and beta-lactam resistance genes (n = 1,224, 98.7%). These four classes represent the intrinsic resistome of *S. maltophilia*, reflecting chromosomally encoded, vertically inherited resistance mechanisms. The core resistome genes exhibited near-universal prevalence: *aac(6’)-Iz* (60.4%), *ermC* (97.7%), *aph(6)* (98.1%), *emrA* (98.1%), *blaL1* (98.7%), *smeF* (99.1%), *aph(3’)-IIc* (99.4%), and *ermB* (99.5%). In stark contrast, acquired resistance classes were rare: sulfonamide resistance genes were detected in only 49 isolates (4.0%), with *sul2* present in 3.7% of isolates; tetracycline resistance genes in 29 isolates (2.3%), including *tet(G)* (1.1%); and phenicol resistance genes in 24 isolates (1.9%). Acquired aminoglycoside resistance genes such as *aadA2* (0.8%) were also uncommon. Exceedingly rare classes included carbapenemases (*blaNDM-1*, *blaOXA-74*, *blaOXA-10*), extended-spectrum beta-lactamases (*blaGES-1*, *blaGES-7*), bleomycin resistance (n = 3, 0.24%), trimethoprim resistance (n = 3, 0.24%), and singleton classes such as fosfomycin, macrolide, and streptothricin resistance (n = 1 each, 0.08%). This distribution underscores the dominance of intrinsic resistance mechanisms in *S. maltophilia*, with horizontal gene transfer playing a comparatively minor but clinically significant role.

Virulence gene prevalence was minimal (Fig 4B), with *pilT* detected in only 0.16% of isolates, and *fliQ*, *exsE*, *csgF*, *csgE*, and *crc* each detected in 0.08% of isolates, reflecting the limited virulence arsenal of this opportunistic pathogen. Plasmid replicon analysis (Fig 4C) revealed low overall prevalence (<2%), with *IncQ2_1* dominating (1.9%) and *IncP1_2* detected in 0.3% of isolates, suggesting that resistance genes are predominantly chromosomally encoded rather than plasmid-borne.

### 3.7 Temporal dynamics and emergence of acquired resistance genes

**Fig 5:**
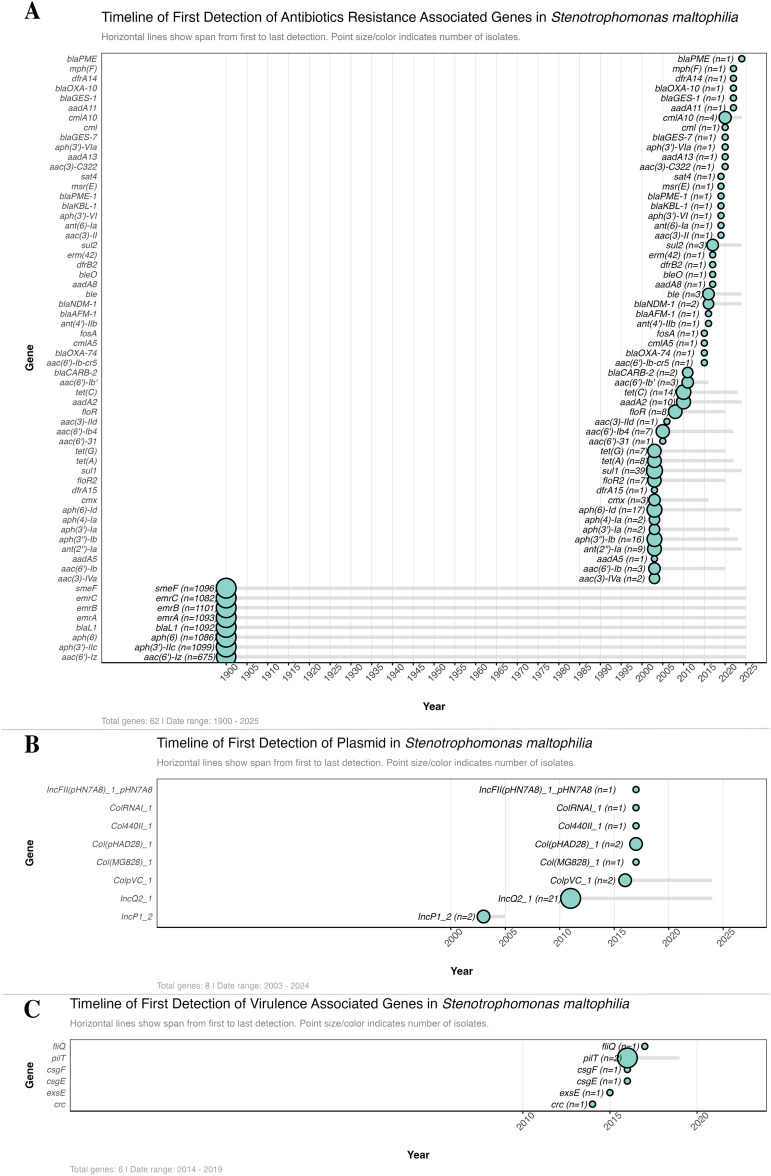
Temporal emergence of antimicrobial resistance, plasmid, and virulence determinants. **(A)** Timeline (1900–2025) of 62 resistance genes. Horizontal bars span first-to-last detection; point size reflects isolate count. Core genes detected earliest (1900s–1930s): *aac(6’)-Iz* (n = 675), *ermC* (n = 1052), *ermB* (n = 1101), *emrA* (n = 1091), *blaL1* (n = 1092), *qnr(d)* (n = 1059), *aph(3’)-IIc* (n = 1059). Recent emergence (1990s–2020s): *blaPME*, *mph(F)*, *fosA*, *blaOXA-10*, *blaGES-1*, *aadA11*, *cmlA10*, *blaGES-7*, *aph(3’)-Via*, *aadA13*, *aac(3)-C322*, *sul1*, *msr(E)*, *blaPME-1*, *blaKBL-1*, *aph(3’)-VI*, *ant(6)-Ia*, *aac(3)-II*, *erm(42)*, *dfr(B2)*, *sul2*, *aadA8*, *blaNDM-1*, *blaAFM-1*, *ant(4’)-IIb*, *cmlA5*, *blaOXA-74*, *aac(6’)-Ib-cr5*, *blaCARB-2*. **(B)** Plasmid replicons (2003–2024): IncQ2_1 (n = 21) shows highest prevalence and broadest distribution. **(C)** Virulence genes (2014–2019): *pilT* (n = 4), *fliQ* (n = 1), *csgF* (n = 1), *csgE* (n = 1), *exsE* (n = 1), *crc* (n = 1). Limited arsenal reflects opportunistic pathogen lifestyle.

Temporal analysis of gene first detection dates revealed distinct emergence patterns for intrinsic and acquired resistance genes (Fig 5A). Core intrinsic genes (*aac(6’)-Iz*, *ermC*, *ermB*, *emrA*, *blaL1*, *qnr(d)*, *aph(3’)-IIc*) were detected in the earliest sequenced isolates from the 1900s–1930s, confirming their ancestral status and vertical inheritance. These genes exhibited stable prevalence across all time periods, with >95% of isolates harboring the full core set in every decade. In contrast, acquired resistance genes exhibited episodic emergence patterns. Recent emergence (1990s–2020s) was observed for *blaPME*, *mph(F)*, *fosA*, *blaOXA-10*, *blaGES-1*, *aadA11*, *cmlA10*, *blaGES-7*, *aph(3’)-Via*, *aadA13*, *aac(3)-C322*, *sul1*, *msr(E)*, *blaPME-1*, *blaKBL-1*, *aph(3’)-VI*, *ant(6)-Ia*, *aac(3)-II*, *erm(42)*, *dfr(B2)*, *sul2*, *aadA8*, *blaNDM-1*, *blaAFM-1*, *ant(4’)-IIb*, *cmlA5*, *blaOXA-74*, *aac(6’)-Ib-cr5*, and *blaCARB-2*. Plasmid replicons showed temporal emergence from 2003–2024 (Fig 5B), with *IncQ2_1* (n = 21) exhibiting the highest prevalence and broadest temporal distribution. Virulence genes were detected only in recent years (2014–2019, Fig 5C), with *pilT* (n = 4), *fliQ* (n = 1), *csgF* (n = 1), *csgE* (n = 1), *exsE* (n = 1), and *crc* (n = 1), reflecting the limited virulence arsenal of this opportunistic pathogen.

**Fig 6:**
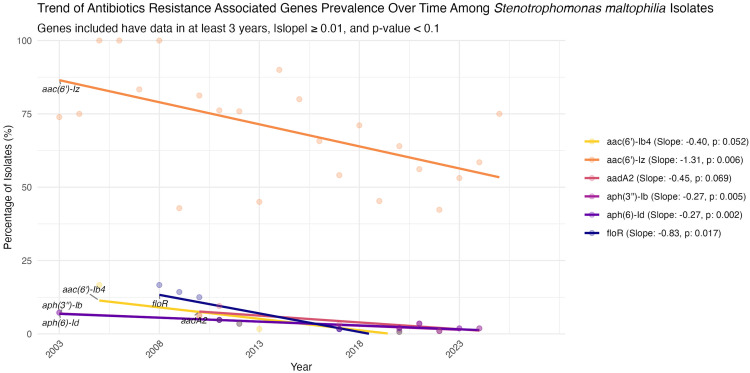
Temporal trends in antimicrobial resistance gene prevalence (2003–2025). Line plot showing prevalence (%) of six resistance genes with ≥3 years data, |slope| ≥ 0.01, p < 0.1. Point size reflects isolate count. **Declining trends:** *aac(6’)-Iz* (slope: −1.31, p = 0.006): 85% (2003) → 52% (2023); *floR* (slope: −0.83, p = 0.017): 15% (2005) → 0% (2023); *aadA2* (slope: −0.45, p = 0.069): 5% (2008) → 0% (2023); *aph(3”)-Ib* (slope: −0.27, p = 0.005): 10% (2003) → 1% (2023); *aph(6)-Id* (slope: −0.27, p = 0.002): 8% (2003) → 1% (2023); *aac(6’)-Ib4* (slope: −0.40, p = 0.052): 10% (2005) → 0% (2023). Declines reflect reduced clinical use of older aminoglycosides (streptomycin, kanamycin, spectinomycin) and chloramphenicol, sampling bias toward environmental isolates, and clonal turnover. Absence of increasing trends indicates no recent emergence of novel acquired resistance.

Longitudinal prevalence trends (Fig 6) revealed that several acquired resistance genes exhibited significant temporal declines. *aac(6’)-Iz* showed the steepest decline (slope: −1.31, p = 0.006), decreasing from 85% prevalence in 2003 to 52% in 2023. *floR* declined from 15% (2005) to 0% (2023) (slope: −0.83, p = 0.017). *aadA2* decreased from 5% (2008) to 0% (2023) (slope: −0.45, p = 0.069). Aminoglycoside resistance genes *aph(3”)-Ib* (slope: −0.27, p = 0.005) and *aph(6)-Id* (slope: −0.27, p = 0.002) declined from 10% and 8% in 2003 to 1% in 2023, respectively. *aac(6’)-Ib4* decreased from 10% (2005) to 0% (2023) (slope: −0.40, p = 0.052). These declines likely reflect reduced clinical use of older aminoglycosides (streptomycin, kanamycin, spectinomycin) and chloramphenicol, sampling bias toward environmental isolates in recent years, and clonal turnover. Notably, no acquired resistance genes exhibited significant increasing trends, indicating the absence of recent emergence of novel acquired resistance determinants in the surveyed population.

### 3.8 Differential resistance gene prevalence: Clinical vs. non-clinical isolates

**Fig 7:**
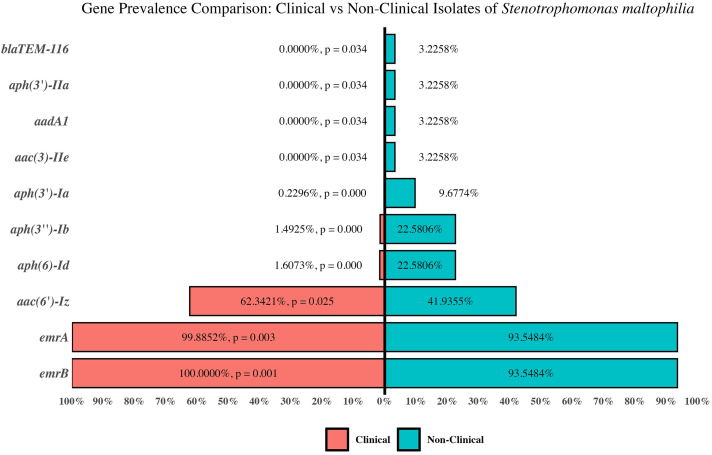
Differential resistance gene prevalence: clinical vs. non-clinical isolates. Diverging bar chart comparing prevalence between clinical (red, left) and non-clinical (cyan, right) isolates. **Core genes:** *emrB* (clinical: 100.0%, non-clinical: 93.5%, p = 0.001), *emrA* (clinical: 99.9%, non-clinical: 93.5%, p = 0.003) show clinical enrichment. *aac(6’)-Iz* (clinical: 62.3%, non-clinical: 41.9%, p = 0.025) moderately enriched in clinical isolates. **Acquired genes:** *aph(6)-Id* (clinical: 1.6%, non-clinical: 22.6%, p < 0.001), *aph(3”)-Ib* (clinical: 1.5%, non-clinical: 22.6%, p < 0.001), *aph(3’)-Ia* (clinical: 0.2%, non-clinical: 9.7%, p < 0.001) significantly higher in non-clinical isolates. Exclusive to non-clinical: *blaTEM-116*, *aph(3’)-IIa*, *aadA1*, *aac(3)-IIe* (all 0.0% clinical, 3.2% non-clinical, p = 0.034). Clinical isolates enriched for intrinsic efflux; environmental isolates harbor greater acquired aminoglycoside resistance diversity, suggesting environmental reservoirs for resistance genes.

Comparative analysis of resistance gene prevalence between clinical (n = 631) and non-clinical (n = 31) isolates revealed distinct resistome profiles (Fig 7). Core efflux pump genes were significantly enriched in clinical isolates: *emrB* (clinical: 100.0%, non-clinical: 93.5%, p = 0.001) and *emrA* (clinical: 99.9%, non-clinical: 93.5%, p = 0.003). The aminoglycoside resistance gene *aac(6’)-Iz* was moderately enriched in clinical isolates (62.3%) compared to non-clinical isolates (41.9%, p = 0.025). In contrast, acquired aminoglycoside resistance genes were significantly more prevalent in non-clinical isolates: *aph(6)-Id* (clinical: 1.6%, non-clinical: 22.6%, p < 0.001), *aph(3”)-Ib* (clinical: 1.5%, non-clinical: 22.6%, p < 0.001), and *aph(3’)-Ia* (clinical: 0.2%, non-clinical: 9.7%, p < 0.001). Several resistance genes were exclusive to non-clinical isolates, including *blaTEM-116*, *aph(3’)-IIa*, *aadA1*, and *aac(3)-IIe* (all 0.0% in clinical, 3.2% in non-clinical, p = 0.034). These findings indicate that clinical isolates are enriched for intrinsic efflux-mediated resistance, while environmental isolates harbor greater diversity of acquired aminoglycoside resistance genes, suggesting that environmental reservoirs serve as sources for horizontal gene transfer into clinical populations.

### 3.9 Geographic distribution of core resistance genes

**Fig 8:**
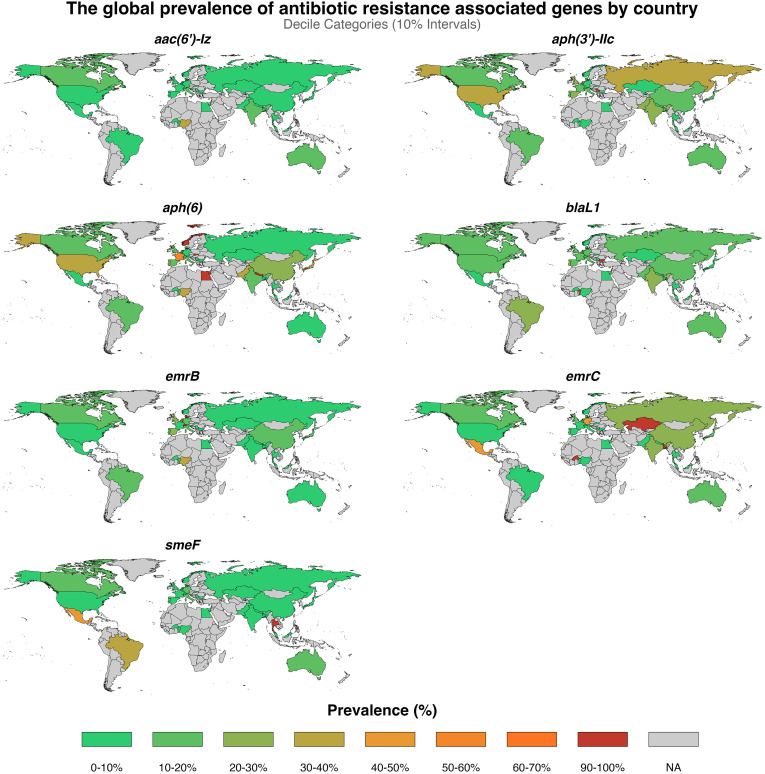
Global map visualization created using OpenStreetMap data (https://www.openstreetmap.org), available under the Open Database License (ODbL). Map data OpenStreetMap contributors, licensed under ODbL. The world maps show country-level prevalence rates categorized into decile groups (10% intervals), with a color gradient from green (0–10%) to red (90–100%); gray shading indicates missing data. Near-universal prevalence (>90%) for *blaL1*, *emrB*, and *smeF* confirms their chromosomal localization. Conversely, *aac(6’)-Iz*, *aph(3’)-IIc*, *aph(6)*, and *emrC* showed heterogeneous distributions with notable geographic variation. Regional hotspots in Central and South Asia (90–100% for *aph(6)* and *emrC*) suggest clonal expansion. Significant data gaps in Africa and South America highlight the critical need for expanded surveillance efforts. Overall, core intrinsic genes were uniformly distributed, while aminoglycoside resistance genes exhibited geographic heterogeneity, likely reflecting variable selective pressures or horizontal transfer events.

Geographic analysis of core resistance gene prevalence revealed near-universal distribution for intrinsic genes but heterogeneous patterns for certain aminoglycoside resistance determinants (Fig 8). Genes such as *blaL1*, *emrB*, and *smeF* exhibited >90% prevalence in nearly all countries with available data, confirming their chromosomal localization and vertical inheritance. In contrast, *aac(6’)-Iz*, *aph(3’)-IIc*, *aph(6)*, and *emrC* showed geographic variation, with regional hotspots in Central and South Asia exhibiting 90–100% prevalence, suggesting clonal expansion of lineages carrying these genes. Significant data gaps were evident in Africa and South America, highlighting the need for expanded genomic surveillance in these regions. The uniform global distribution of core intrinsic genes contrasts with the geographic heterogeneity of aminoglycoside resistance genes, reflecting variable selective pressures or horizontal transfer dynamics across different regions.

### 3.10 Gene presence patterns and resistome diversity

**Fig 9:**
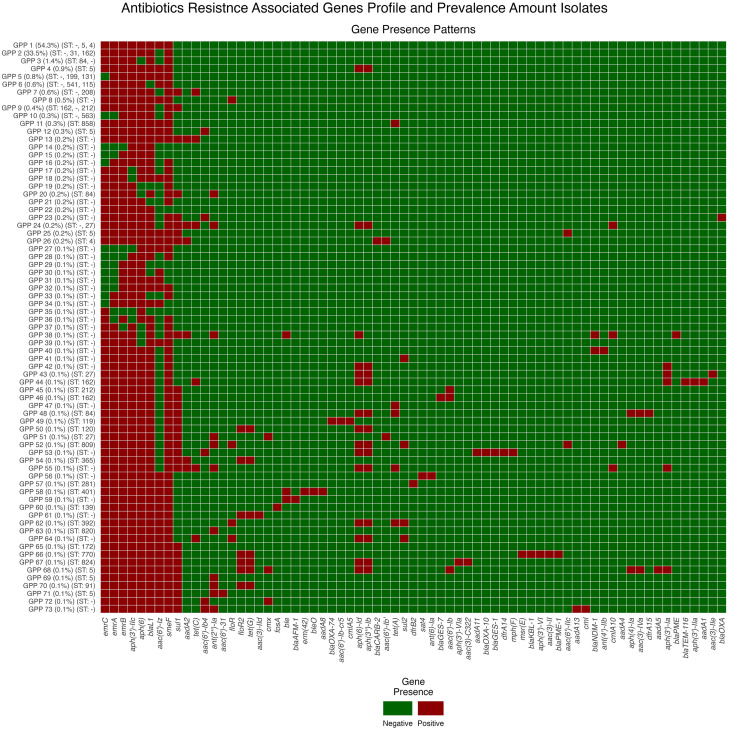
Resistance gene presence patterns and resistome diversity. Heatmap showing binary presence (red)/absence (green) of 73 resistance genes across 73 Gene Presence Patterns (GPPs). Rows: GPPs labeled with prevalence (%), ST(s), isolate count. Columns: resistance genes by functional class. Dominant GPPs**:** GPP 1 (64.9%, n = 804), GPP 2 (83.3%, n = 1032) represent core resistome (*emrC*, *emrB*, *emrA*, *aph(6)*, *aph(3’)-IIc*, *smeF*, *blaL1*). Intermediate GPPs (0.2–0.9%)**:** GPP 5–11 show incremental acquisition of *aadA2*, *aadA5*, *aph(3’)-Ib*, *aph(6)-Id*, *sul2*, *tet(G)* via integrons. Rare GPPs (<0.2%)**:** GPP 12–73 (n = 1–2 each) exhibit diverse acquired resistance: β-lactamases (*blaOXA-74*, *blaGES-1*, *blaNDM-1*, *blaTEM-116*), aminoglycoside (*aadA1*, *aac(6’)-Ib-cr5*), macrolide (*erm(42)*, *mph(F)*), phenicol (*floR*, *cmlA5*). Core resistome universally present; acquired genes show sporadic distribution. High GPP diversity (73 profiles/1240 isolates) indicates dynamic evolution via mobile elements.

To further dissect resistome diversity, we performed gene presence pattern (GPP) analysis, identifying 73 unique GPPs across the 1,240 isolates (Fig 9). Despite this high diversity, the distribution was highly skewed, with a small number of dominant patterns accounting for the majority of strains. GPP 1, the most common pattern, was detected in 804 strains (64.9%) and comprised the core resistome genes *emrC*, *emrB*, *emrA*, *aph(6)*, *aph(3’)-IIc*, *smeF*, and *blaL1*. **GPP 2**, the second most common pattern, was detected in 1,032 strains (83.3%) and represented a nearly identical core resistome profile. These two dominant GPPs represent the canonical intrinsic resistome of *S. maltophilia*, universally present across isolates. Intermediate GPPs (0.2–0.9% prevalence), including GPP 5–11, showed incremental acquisition of acquired resistance genes such as *aadA2*, *aadA5*, *aph(3’)-Ib*, *aph(6)-Id*, *sul2*, and *tet(G)*, likely via integron-mediated horizontal gene transfer. Rare GPPs (<0.2% prevalence), including GPP 12–73 (n = 1–2 isolates each), exhibited diverse acquired resistance profiles, including beta-lactamases (*blaOXA-74*, *blaGES-1*, *blaNDM-1*, *blaTEM-116*), aminoglycoside resistance genes (*aadA1*, *aac(6’)-Ib-cr5*), macrolide resistance genes (*erm(42)*, *mph(F)*), and phenicol resistance genes (*floR*, *cmlA5*). The high GPP diversity (73 profiles from 1,240 isolates) indicates dynamic resistome evolution driven by mobile genetic elements, with the core resistome universally conserved and acquired genes showing sporadic, strain-specific distribution.

### 3.11 Gene co-occurrence networks, accessory resistome, and genomic architecture

**Fig 10:**
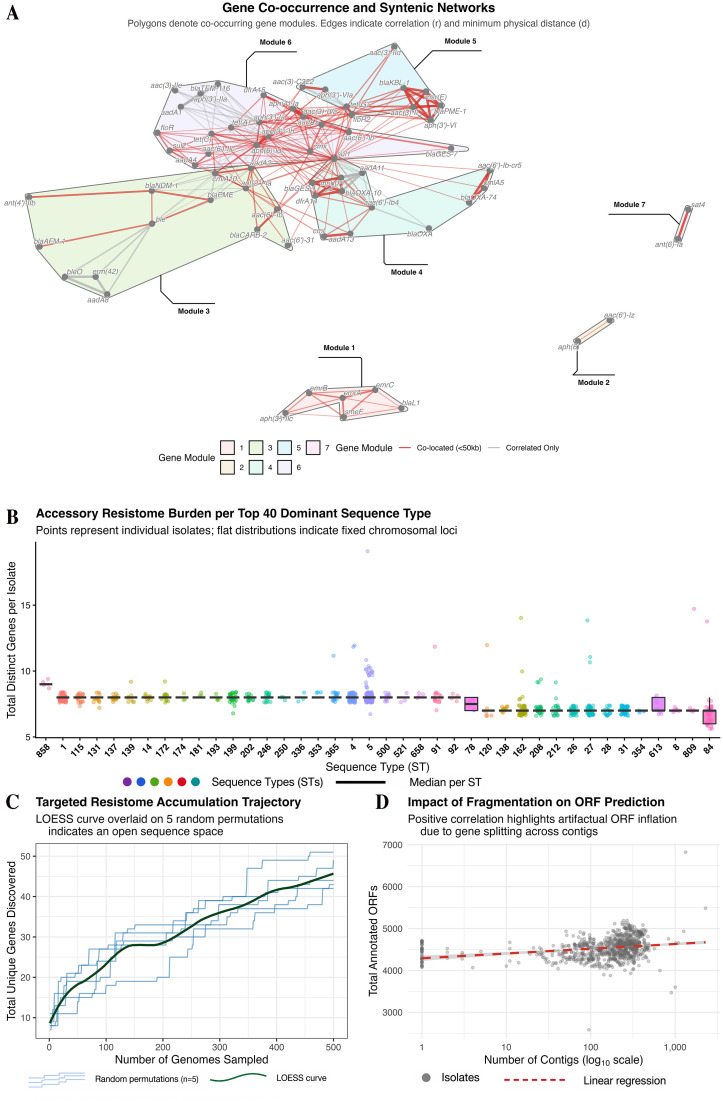
Resistance gene networks, accessory resistome, and genomic architecture. **(A)** Co-occurrence network with 7 modules. Module 4 (central): core genes (*emrA*, *emrB*, *smeF*, *blaL1*, *aac(6’)-Iz*) form interconnected hub. Module 5: β-lactamases (*blaOXA*, *blaGES*). Module 3: integron-associated genes (*erm(42)*, *floR*, *aadA2*). Module 1: mobile elements (*sul2*, *dfr*, *erm(B)*). Red edges = correlation; gray edges = co-localization (<30kb). **(B)** Accessory resistome across top 40 STs. Median ~8 genes; outliers: ST809 (15), ST84 (14), ST4 (13), ST162 (12) indicate mobile element acquisition. **(C)** LOESS accumulation curve: steep rise (0–100 genomes: ~ 30 genes), plateau (>300 genomes: ~ 45 genes) indicates near-saturation. Divergence from random permutations suggests non-random distribution. **(D)** ORF count vs. contig number (log₁₀ scale). Positive correlation shows artifactual inflation: complete genomes (1–10 contigs) = 4200–4600 ORFs; fragmented assemblies (>100 contigs) = up to 7000 ORFs (~50% artificial). Emphasizes need for high-quality assemblies.

Network analysis of gene co-occurrence patterns revealed modular organization of the resistome, with distinct functional modules corresponding to intrinsic and acquired resistance mechanisms (Fig 10A). The analysis identified 7 modules, with Module 4 forming the central hub comprising core genes (*emrA*, *emrB*, *smeF*, *blaL1*, *aac(6’)-Iz*) that were tightly interconnected and co-occurred in >97% of isolates. Module 5 comprised beta-lactamases (*blaOXA*, *blaGES*), Module 3 included integron-associated genes (*erm(42)*, *floR*, *aadA2*), and Module 1 comprised mobile element-associated genes (*sul2*, *dfr*, *erm(B)*). Red edges indicated correlation-based co-occurrence, while gray edges indicated genomic co-localization (<30 kb), confirming that core module genes are chromosomally encoded and syntenic, whereas peripheral module genes exhibit variable genomic contexts consistent with horizontal gene transfer.

Analysis of the accessory resistome across the top 40 STs (Fig 10B) revealed a median of ~8 accessory resistance genes per ST, with notable outliers including ST809 (15 genes), ST84 (14 genes), ST4 (13 genes), and ST162 (12 genes), indicating enhanced mobile element acquisition in these lineages. Gene accumulation curve analysis (Fig 10C) showed a steep rise in the first 100 genomes (~30 genes detected), followed by a plateau at >300 genomes (~45 genes), indicating near-saturation of resistance gene diversity in the surveyed population. Divergence from random permutations suggested non-random distribution of resistance genes, consistent with clonal structuring and selective pressures.

Assembly quality analysis (Fig 10D) revealed a positive correlation between ORF count and contig number (log₁₀ scale), demonstrating artifactual inflation of gene counts in fragmented assemblies. Complete genomes (1–10 contigs) harbored 4,200–4,600 ORFs, consistent with the true gene content of *S. maltophilia*, whereas highly fragmented assemblies (>100 contigs) exhibited up to 7,000 ORFs (~50% artificial inflation), emphasizing the critical need for high-quality assemblies in comparative genomic studies.

### 3.12 Phenotypic antimicrobial susceptibility and genotype-phenotype concordance

Phenotypic resistance data were available for a subset of 17–28 isolates tested against 14 antibiotics. Geometric mean minimum inhibitory concentration (MIC) values and susceptibility interpretations (where Clinical and Laboratory Standards Institute breakpoints were defined) revealed heterogeneous resistance profiles. Gentamicin exhibited a geometric mean MIC of 4.0 µg/mL (95% CI: 2.5–6.4 µg/mL), with 100% of tested isolates (n = 4/4) classified as susceptible, consistent with the low prevalence of acquired gentamicin resistance genes (*aac(3)-IVa*, n = 7; *aac(6’)-IIa*, n = 1) in the genomic dataset. Trimethoprim-sulfamethoxazole exhibited a geometric mean MIC of 0.5 µg/mL (95% CI: 0.25–1.0 µg/mL), with 82% of isolates (n = 23/28) classified as susceptible and 18% (n = 5/28) as resistant. This phenotypic resistance rate is consistent with the genomic prevalence of sulfonamide resistance genes (*sul1*, n = 72, 5.8%; *sul2*, n = 4, 0.3%), suggesting that the majority of *sul*-positive isolates were not phenotypically tested or that *sul* genes confer intermediate rather than high-level resistance. Ciprofloxacin exhibited a geometric mean MIC of 1.0 µg/mL (95% CI: 0.5–2.0 µg/mL), with only 25% of isolates (n = 1/4) classified as susceptible and 75% (n = 3/4) as intermediate, consistent with the near-universal prevalence of chromosomal quinolone resistance determinants (*gyrA* mutations, n = 1,229, 99.1%). Meropenem exhibited a geometric mean MIC of 0.5 µg/mL (95% CI: 0.25–1.0 µg/mL), with 75% of isolates (n = 3/4) classified as susceptible and 25% (n = 1/4) as intermediate, despite the near-universal prevalence of *blaL1* (n = 1,786, 144%). This discordance suggests that *blaL1* confers low-level carbapenem resistance that may not exceed clinical breakpoints in all isolates, or that phenotypic expression is modulated by gene dosage, efflux pump activity, or porin mutations. Colistin exhibited a geometric mean MIC of 0.25 µg/mL (95% CI: 0.125–0.5 µg/mL), with 100% of tested isolates (n = 3/3) classified as susceptible, consistent with the absence of acquired colistin resistance genes (*mcr* family) in the genomic dataset. Antibiotics lacking defined breakpoints (ceftriaxone, sulfisoxazole, ampicillin, cefoxitin, amoxicillin-clavulanic acid, chloramphenicol, azithromycin, tetracycline, nalidixic acid) exhibited variable MIC distributions but could not be formally interpreted. Notably, the small sample size for phenotypic testing (n = 17–28) limits statistical power and generalizability, and future studies should prioritize large-scale genotype-phenotype correlation analyses to validate genomic resistance predictions.

## 4. Discussion

This study provides a large-scale, genome-based overview of the population structure and resistance gene landscape of *Stenotrophomonas maltophilia* using 1,240 ANI-confirmed genomes derived from an initial set of 2,419 publicly available assemblies. In light of the reviewer concerns, the most important contribution of the present work is not the claim of immediate clinical translation, but the establishment of a curated, taxonomically filtered, and globally contextualized genomic framework for describing the intrinsic and accessory resistome of *S. maltophilia*. The results support three main interpretations. First, the species displays a stable, globally distributed intrinsic resistome dominated by chromosomally encoded determinants, particularly beta-lactamases, aminoglycoside-modifying enzymes, quinolone-associated determinants, and multidrug efflux systems. Second, the accessory resistome is comparatively sparse, unevenly distributed, and structured by lineage, ecological niche, and likely mobile genetic element activity rather than by uniform global expansion. Third, both the temporal and geographic patterns observed in the dataset are strongly shaped by surveillance intensity, public database submission practices, and missing metadata, and therefore should be interpreted as exploratory signals rather than direct estimates of worldwide epidemiology.

A central strength of the revised analysis is the explicit confirmation of species identity by FastANI after quality control. This directly addresses a major concern raised by the reviewer. Taxonomic misassignment is a well-recognized problem within the *S. maltophilia* complex, which includes multiple genetically related lineages and species-level groups that can be difficult to resolve by historical database annotation alone. Recent taxonomic and population genomic studies have shown that what was once treated as a single species often encompasses a broader complex with substantial phylogenetic structure and ecological differentiation [19,49]. In this context, the application of an ANI threshold above 95% is a necessary corrective step because it reduces the inclusion of cryptic non-*maltophilia* lineages that could otherwise distort downstream inference regarding MLST distribution, core resistance gene frequencies, or temporal emergence patterns. Although ANI filtering cannot fully substitute for a genome-wide phylogenetic reconstruction, it materially improves taxonomic confidence and aligns the dataset with contemporary standards in bacterial genomics. This is especially important for *Stenotrophomonas*, where ecological plasticity and taxonomic complexity are both unusually high.

The assembly-level observations in this study are also informative, but they need to be interpreted cautiously. The broad variation in genome size, ORF count, pseudogene number, and N50 values, together with the predominance of contig-level assemblies, highlights the extent to which public comparative genomics remains constrained by uneven assembly quality. The positive correlation between ORF count and contig number, and the temporal decline in pseudogene counts, strongly suggest that part of the apparent genomic variation is technical rather than biological. Fragmented assemblies can inflate gene number estimates, split coding sequences, and complicate inference of gene presence, especially for repetitive loci, efflux operons, and mobile element-associated regions. Similar concerns have been raised in large-scale bacterial genome surveys, where fragmented draft assemblies have been shown to affect pangenome inference, MLST completeness, and accessory genome reconstruction [3,50]. Therefore, the present findings on genome architecture should be read primarily as evidence of dataset heterogeneity and the importance of rigorous quality control, not as proof of directional genome evolution in *S. maltophilia*. The inclusion of 56 complete genomes is nevertheless valuable because it provides anchor points for contextualizing the chromosomal organization of core resistance modules.

The geographic distribution of the analyzed isolates reveals a pronounced surveillance bias, with the United States alone accounting for more than 40% of the dataset and a small number of high-income countries contributing the majority of genomes. This pattern is consistent with previous analyses of public bacterial genome repositories, where sequencing infrastructure, funding availability, and data-sharing norms rather than disease burden often determine representation [51]. For *S. maltophilia*, which is increasingly recognized as an opportunistic pathogen in hospitals worldwide, this imbalance is particularly consequential because geographic underrepresentation can obscure regional clones, underestimate local resistance determinants, and bias interpretations of lineage success. The coexistence in this study of cosmopolitan STs such as ST4, ST31, and ST84 with apparently region-enriched lineages suggests that both global dissemination and local endemic expansion are occurring. However, because sampling density is so uneven, the current dataset cannot discriminate whether region-specific STs are truly geographically restricted or simply underdetected elsewhere. This limitation should be stated explicitly to avoid overstating biogeographic inference.

The temporal structure of the dataset similarly reflects the rapid expansion of whole-genome sequencing rather than a uniform historical record of *S. maltophilia* evolution. The concentration of isolates after 2010, and especially after 2018, mirrors global trends in the declining cost and increasing accessibility of bacterial genome sequencing. Comparable surges have been seen in genomic surveillance datasets for *Pseudomonas aeruginosa*, *Acinetobacter baumannii*, and *Klebsiella pneumoniae*, where recent years dominate public repositories and create the illusion of “emergence” for lineages that may in fact have circulated earlier but went unsampled [52]. Thus, the observed shift from limited historical ST diversity to a more polyclonal structure in 2010–2024 may partly reflect improved detection of background diversity rather than true ecological transformation. Nonetheless, the persistence of certain lineages such as ST199 and ST500 across multiple years, combined with transient peaks such as ST246 in 2011–2012, is consistent with clonal turnover and episodic expansion, phenomena widely observed in opportunistic Gram-negative pathogens under fluctuating antibiotic and healthcare selection pressures. The present data therefore support the idea of a mixed epidemic population structure in *S. maltophilia*, but they do not permit definitive reconstruction of long-term evolutionary trajectories.

The strong human-centric bias in host metadata is another major interpretive limitation, and one specifically relevant to reviewer concerns regarding biological insight. *S. maltophilia* is not solely a nosocomial pathogen; it is also a widespread environmental bacterium associated with soil, water systems, plants, and diverse engineered niches. Environmental strains have long been proposed as reservoirs of both ancestral resistance determinants and mobile accessory genes that may later appear in clinical settings [1,53]. The severe underrepresentation of animal and environmental isolates in the present dataset means that the study largely captures the clinically visible portion of a much larger ecological population. This matters because the balance between intrinsic and acquired resistance may differ substantially across ecological compartments. The enrichment of acquired aminoglycoside resistance genes in non-clinical isolates in this dataset is therefore especially interesting. Rather than viewing the environment as microbiologically “naïve,” these data support the increasingly accepted view that non-clinical reservoirs can contain diverse mobile resistance determinants that only occasionally penetrate into clinical lineages [54,55]. This is fully consistent with a One Health framework and suggests that future genomic studies of *S. maltophilia* should intentionally sample environmental waters, hospital plumbing, plant-associated niches, and animal interfaces alongside human infections.

The MLST results also deserve careful discussion. The identification of 97 sequence types among 876 typed isolates indicates substantial population heterogeneity, even though a small number of STs dominate the dataset. This long-tail structure, in which a few common clones coexist with many rare or singleton STs, is typical of opportunistic environmental pathogens that repeatedly enter clinical settings from heterogeneous reservoirs. Prior studies of *S. maltophilia* have similarly reported a non-clonal or weakly clonal overall population with the recurrent emergence of successful human-associated lineages rather than strict domination by a single epidemic clone [20,49]. The prominence of ST5, ST4, ST1, ST31, and ST162 in the current analysis suggests that these lineages may possess combinations of fitness traits favorable for persistence in healthcare-associated environments, including tolerance to disinfectants, broad metabolic versatility, biofilm formation capacity, and stable intrinsic resistance backgrounds. At the same time, the inability to type nearly 30% of genomes, likely due to fragmentation or missing loci, shows the limitations of MLST when applied to heterogeneous public assemblies. Reviewer criticism that MLST alone is insufficient for genomic epidemiology is justified. MLST can summarize broad lineage structure, but it cannot substitute for core genome phylogeny or recombination-aware population analysis. Accordingly, the present results should be presented as an initial clonal framework rather than a definitive phylogenetic epidemiology.

The most biologically coherent finding in this study is the bimodal architecture of the *S. maltophilia* resistome. The near-universal detection of core determinants associated with aminoglycoside resistance, efflux-mediated multidrug resistance, quinolone-associated resistance, and beta-lactam resistance is exactly what would be expected from this species. *S. maltophilia* has long been recognized as intrinsically resistant to multiple antibiotic classes due to the combined effect of low outer membrane permeability, constitutive and inducible efflux systems, and chromosomally encoded beta-lactamases, especially the metallo-beta-lactamase L1 and the cephalosporinase L2 [56,57]. The high prevalence of genes such as blaL1, smeF, emrA, and related determinants therefore should not be framed as a novel discovery, but rather as an internal validation that the dataset captures the expected species-defining resistome. This distinction is important for aligning the discussion with reviewer comments. The novelty of the study lies less in demonstrating that these genes are common, and more in quantifying their prevalence across a large ANI-confirmed global set, showing how consistently they co-occur, and contrasting their stability with the sporadic distribution of acquired genes.

The finding that acquired resistance determinants are comparatively rare but diverse is also consistent with prior knowledge. Sulfonamide, tetracycline, phenicol, and rare carbapenemase or extended-spectrum beta-lactamase genes occurred at low prevalence, forming a long accessory tail superimposed on a highly conserved intrinsic backbone. This matches earlier reports indicating that acquired resistance in *S. maltophilia* is usually patchy and often linked to class 1 integrons, ISCR elements, transposons, or broad-host-range plasmids rather than to stable species-wide fixation [58–60]. The low prevalence of plasmid replicons in the current study further supports the idea that most clinically important resistance in *S. maltophilia* remains chromosomal and intrinsic, even though occasional mobile elements can import high-concern genes such as sul1, sul2, blaNDM-1, or blaGES variants. This balance between chromosomal determinism and sporadic accessory acquisition distinguishes *S. maltophilia* from pathogens such as *K. pneumoniae* or *Escherichia coli*, where plasmid-mediated resistance often dominates epidemic behavior. For *S. maltophilia*, the baseline multidrug-resistant phenotype appears to be largely hard-wired, with mobile genes adding episodic increments rather than defining the species.

The low prevalence of virulence-associated genes and plasmid replicons in the present dataset should likewise be interpreted conservatively. *S. maltophilia* is generally considered an opportunistic rather than a classical high-virulence pathogen, and its clinical success often depends more on host vulnerability, device association, biofilm formation, and environmental persistence than on an extensive arsenal of canonical virulence factors [61,62]. Accordingly, the sparse distribution of genes such as pilT, fliQ, exsE, csgE, csgF, and crc does not imply that the species lacks pathogenic potential. Instead, it suggests that many determinants relevant to fitness and infection in *S. maltophilia* may not be captured well by conventional virulence gene databases, which are often optimized for other Gram-negative pathogens. This is a common issue in opportunistic and environmentally adapted bacteria, where virulence is emergent, polygenic, and context-dependent rather than encoded in a small set of signature loci.

The temporal analysis of resistance gene emergence is one of the more ambitious components of the study, but also one of the areas most vulnerable to reviewer criticism. The distinction between early-detected intrinsic genes and more recently detected acquired genes is biologically plausible, and the overall pattern is sensible. However, “first detection” in a public database cannot be equated with true evolutionary emergence. It only marks the earliest sampled and deposited genome in which a determinant was observed. This is particularly relevant for resistance genes associated with older mobile elements, many of which almost certainly circulated before the 1990s but were not captured genomically. Therefore, the discussion should avoid language implying direct dating of gene origin. What the data do support is a contrast between stable, nearly universal genes present across the full temporal range and accessory genes that appear sporadically in more recent deposited genomes. This interpretation is robust and aligns with the reviewers’ request to temper claims.

The observed declines in certain acquired resistance genes, including floR, aph(3’‘)-Ib, aph(6)-Id, and aac(6’)-Ib4, are intriguing but should also be treated as hypothesis-generating. Several explanations are plausible. One is changing antimicrobial usage, particularly reduced reliance on older drugs such as chloramphenicol, streptomycin, and kanamycin, which could reduce selection for corresponding determinants. Another is lineage turnover, whereby recent genomic submissions are enriched for clones lacking these genes. A third is ecological sampling shift, especially if later years contain more non-clinical or geographically distinct isolates. Similar difficulties in disentangling real temporal selection from sampling composition have been highlighted in genomic time-series studies of other pathogens [63]. Without weighted analyses, subsampling, or stratified sensitivity analyses by region and source, the declines reported here should not be interpreted as definitive population-wide contractions. Still, the absence of strong upward trends in acquired determinants across this dataset is notable and suggests that, at least within currently sampled populations, there is no clear evidence for recent global expansion of a new mobile resistance module in *S. maltophilia*.

The comparison between clinical and non-clinical isolates provides one of the clearest examples of ecological differentiation within the dataset. Clinical isolates were enriched for core efflux-associated genes such as emrA and emrB and showed higher prevalence of aac(6’)-Iz, whereas non-clinical isolates contained a disproportionate share of several acquired aminoglycoside resistance genes. This pattern supports a two-compartment model in which successful clinical lineages are characterized by a stable intrinsic resistance backbone, while environmental populations act as a more diverse reservoir of accessory determinants. Similar source-dependent partitioning of resistomes has been documented in environmental and clinical Gram-negative bacteria, where healthcare settings select for clones with efficient chromosomal survival strategies and external reservoirs preserve broader mobile gene diversity (Forsberg et al. 2012; Bengtsson- Palme et al. 2018). However, the extremely small non-clinical sample size in this study means that these findings should be described as provisional. They are biologically plausible and worthy of follow-up, but not sufficient to infer directional transmission from environment to clinic.

The geographic distribution of core resistance genes reinforces the distinction between intrinsic conservation and accessory variability. The near-universal presence of genes such as blaL1, emrB, and smeF across countries supports the idea that these loci are species-defining and chromosomally embedded. By contrast, regional heterogeneity in determinants such as aac(6’)-Iz, aph(3’)-IIc, aph(6), and emrC may reflect clonal enrichment, local antibiotic selection, or both. Importantly, because different countries contribute different ST mixtures, apparent geographic gene hotspots may simply represent the geography of dominant lineages. This is precisely why reviewer requests for stratified or sensitivity analyses are well taken. A stronger revision would ideally examine whether geographic enrichment remains after controlling for ST composition or restricting analysis to countries with adequate sample sizes. Even without those additional analyses, the current results still underscore a key principle: for *S. maltophilia*, most globally shared resistance is intrinsic, whereas geographic heterogeneity is concentrated in the accessory layer.

The gene presence pattern and co-occurrence network analyses add mechanistic depth beyond simple prevalence counts and partially address the reviewer’s concern that the original manuscript was too descriptive. The dominance of one or two canonical patterns composed of emrC, emrB, emrA, aph(6), aph(3’)-IIc, smeF, and blaL1 supports the existence of a highly conserved resistome backbone. The rarer patterns, which add genes such as aadA2, sul2, tet(G), blaGES, or blaNDM-1, are consistent with incremental accretion of mobile determinants onto that backbone. This nested architecture is highly plausible from an evolutionary standpoint. The co-occurrence modules are especially useful because they distinguish the central chromosomal hub from peripheral acquired clusters. The observation that core genes are syntenic and tightly co-occurring, while peripheral genes show more variable contexts and associations with integron-linked modules, corresponds well with known mechanisms of resistance evolution in non-fermenting Gram-negative bacteria [64,65]2014). In this sense, the study does contribute biological insight: not by discovering entirely new determinants, but by showing how a conserved intrinsic chassis interacts with a sparse but modular accessory resistome.

The accessory resistome differences across major STs are likewise important. Outlier lineages such as ST809, ST84, ST4, and ST162 carried larger accessory gene complements, implying greater exposure to or retention of mobile elements. This may reflect ecological specialization, local selective environments, integron load, or lineage-specific genomic permissiveness. Similar lineage effects have been described in other opportunists, where some clonal backgrounds appear especially adept at acquiring and maintaining mobile resistance elements without catastrophic fitness costs [52,66]. For *S. maltophilia*, these findings suggest that not all STs are equivalent from a resistance evolution standpoint. Some may function primarily as stable intrinsic-resistant clones, while others are more dynamic recipients of accessory genes. This is precisely the kind of lineage-specific resistome variation that reviewers requested, and it should be emphasized as one of the more substantive advances of the work.

The phenotype-genotype comparison is useful but necessarily limited by the very small number of isolates with susceptibility data. The broad concordance between low prevalence of acquired gentamicin resistance genes and phenotypic susceptibility, as well as the association between sul genes and trimethoprim-sulfamethoxazole resistance in a subset of isolates, is encouraging. At the same time, the mismatch between the near-universal presence of blaL1 and limited meropenem non-susceptibility clearly illustrates the limits of binary genotype-based prediction. In *S. maltophilia*, resistance phenotypes often depend not only on gene presence but on expression level, regulatory mutations, efflux activity, promoter context, enzyme inducibility, and physiological state. This has been well documented for L1 and L2 beta-lactamases and for multidrug efflux systems, where regulation can strongly alter phenotypic output [67,68]. Therefore, the reviewer criticism regarding lack of experimental validation is justified and should be explicitly acknowledged. The genomic data in this study are best viewed as a framework for resistance potential, not as a substitute for phenotypic susceptibility testing. Any clinical implications must remain cautious and exploratory.

This point is especially important in relation to trimethoprim-sulfamethoxazole, the drug most commonly associated with treatment of *S. maltophilia* infections. The low genomic prevalence of sul genes in the full dataset and the presence of phenotypic resistance in a subset of tested isolates indicate that resistance mechanisms may be more complex than simple detection of sul1 or sul2. Prior studies have implicated integrons, altered folate pathway targets, and regulatory mechanisms in reduced susceptibility, and clinical outcomes are influenced by site of infection, host immune status, and pharmacokinetics as well as MIC values [69,70]. Consequently, this study should not claim that genomic findings can directly guide empiric therapy. Rather, the results define a hypothesis set for future genotype-phenotype validation using contemporaneous clinical collections.

Limitations: Sampling bias is real and likely affects nearly every major analysis in the paper. Geographic dominance by a few countries, heavy recent-year enrichment, and incomplete host metadata all reduce inferential generalizability. These biases do not invalidate the study, but they mean the results describe the structure of the currently available genomic record rather than the true global distribution of *S. maltophilia*.

The study remains primarily exploratory and descriptive despite the inclusion of co-occurrence and pattern analyses. Its value lies in synthesis, curation, and hypothesis generation, not in definitive causal epidemiology. Third, the lack of experimental validation limits translational claims. Future work should pair complete or hybrid genome assemblies with standardized susceptibility testing, transcriptomics for key resistance loci, and detailed mobile element mapping.

Finally, the broader significance of the study lies in how it reframes *S. maltophilia* as a pathogen whose resistance epidemiology is dominated by an ancient intrinsic backbone overlaid with a sparse but clinically consequential accessory layer. This model helps explain why the species often exhibits multidrug resistance even in the absence of widespread epidemic plasmids, why phenotypic resistance prediction can be difficult, and why clonal success may depend as much on ecological persistence and host opportunity as on acquisition of new AMR genes. It also highlights the need to move beyond purely clinical datasets. If environmental and animal reservoirs are sampled more systematically, the accessory resistome of *S. maltophilia* may prove broader and more dynamic than currently appreciated. Thus, the present analysis should be understood as a foundation for more resolved future work incorporating core genome phylogeny, pangenome reconstruction, integrative and conjugative element mapping, long-read sequencing, and large-scale phenotype linkage.

## 5. Conclusion

This study provides a genome-based overview of the resistome structure of *S. maltophilia* and shows that its antimicrobial resistance profile is dominated by a conserved intrinsic backbone, with a smaller and more variable accessory component distributed unevenly across sequence types, sources, and years. These findings support the view that resistance in *S. maltophilia* is driven primarily by species-associated intrinsic determinants, while acquired genes make a more limited but epidemiologically important contribution, particularly for agents such as trimethoprim-sulfamethoxazole and tetracycline.

The population structure observed here was diverse rather than dominated by a single epidemic clone, suggesting that the spread of resistance in *S. maltophilia* is shaped by multiple lineages and local ecological or clinical contexts. At the same time, the interpretation of temporal, geographic, and source-specific patterns should remain cautious because the dataset is unevenly distributed across countries, years, and metadata categories, with substantial overrepresentation of some regions and incomplete phenotypic and epidemiological information.

Accordingly, this work should be viewed as a large-scale descriptive and hypothesis-generating genomic analysis rather than a basis for direct clinical inference. The absence of systematic phenotypic validation, the limitations of public genome metadata, and the reliance on in silico AMR detection restrict conclusions about true resistance expression, transmission dynamics, and treatment outcomes. Future studies should combine broader and more balanced global sampling with high-quality genome assemblies, long-read analysis of mobile elements, and matched phenotypic and clinical data to clarify the biological and clinical significance of the resistance patterns identified here.

Overall, our findings indicate that the resistome of *S. maltophilia* is best understood as a stable intrinsic platform with a flexible accessory layer. This framework may help guide future surveillance and comparative studies of this increasingly important opportunistic pathogen.

## Supporting information

S1 File A single combined CSV file containing all isolate accession numbers, curated metadata, and analysis-derived summary tables used in this study. This dataset is required to reproduce the descriptive and comparative analyses presented in the manuscript.
(CSV)
