# Supplementary material for: In silico antimicrobial resistance profiling of 1,240 Stenotrophomonas maltophilia genomes: AMR gene prevalence, temporal evolution, and ST-specific associations
Source: PLoS One. 2026 Jul 9;21(7):e0350669. doi: 10.1371/journal.pone.0350669 (PMC13349159; doi:10.1371/journal.pone.0350669)
Supplement: Attachment — Submitted filename: Response to Reviewers.docx [file pone.0350669.s003.docx]

**Response to Editor and Reviewer**

*We thank the Academic Editor and Reviewer #1 for their careful evaluation of our manuscript and for the constructive comments. We have revised the manuscript extensively to address all concerns. In particular, we clarified that Average Nucleotide Identity (ANI) was used for confirmation of included isolates, we strengthened the temporal regression analysis by applying weighted and unweighted regression approaches and excluding years with fewer than two isolate reports, we expanded the limitations in the Discussion, we tempered statements regarding clinical implications, we added clarification regarding generative AI use, and we updated the manuscript context with relevant landmark studies.*

**Additional Editor Comments:**

The manuscript presents a large-scale genomic analysis of antimicrobial resistance (AMR) in Stenotrophomonas maltophilia, leveraging 2,389 high-quality genomes to investigate AMR gene prevalence, temporal trends, and sequence type (ST)-specific resistance profiles. The study addresses an important gap in understanding the genomic epidemiology of this multidrug-resistant pathogen and contributes valuable insights into intrinsic resistance mechanisms, clonal expansion, and geographic variability. Strengths include the robust dataset, stringent quality-control measures, and transparent reporting of methods. However, significant weaknesses—particularly the reliance on publicly available data, lack of experimental validation, overstated clinical implications, and ethical concerns related to generative AI use—undermine the manuscript’s overall impact. While the findings are biologically plausible and methodologically sound, they fall short of transformative contributions due to unresolved limitations.

**Response:**

*We thank the Editor for this balanced assessment. We have revised the manuscript to better align the scope of our conclusions with the nature of the dataset and the analytical design. Specifically, we now more clearly present the study as a large-scale exploratory genomic analysis rather than a transformative or directly practice-changing investigation. We have also strengthened the manuscript by clarifying species confirmation using ANI, expanding the Discussion of sampling bias and metadata limitations, explicitly acknowledging the lack of experimental validation, tempering clinical interpretation, and adding a transparent Declaration of Generative AI Use with clear author oversight.*

**1. Scientific Rigor:**

**Weaknesses: Sampling biases, uneven metadata availability, and lack of experimental validation for predicted AMR phenotypes reduce confidence in findings.**

**Response:**

*We agree and have addressed these issues in the revised manuscript. We now explicitly acknowledge that the use of publicly available genomes introduces geographic, temporal, and source-related sampling biases, as well as uneven metadata completeness. To improve robustness of the temporal analysis, we used both weighted and unweighted regression models and excluded years with fewer than two isolate reports, thereby reducing distortion from sparsely represented years. In addition, we expanded the Discussion to state clearly that predicted AMR profiles are based on in silico gene detection and do not substitute for phenotypic susceptibility testing. We now frame these results as hypothesis-generating and suitable for future validation rather than definitive evidence of expressed resistance phenotypes.*

**2. Methodological Transparency:**

**Weaknesses: Missing details on raw data availability (e.g., accession numbers, scripts) and parameters used for analyses hinder reproducibility.**

**Response:**

*We thank the Editor for this comment. We have revised the manuscript to ensure that all accession numbers and related identifiers are fully provided in the Supplementary File, and that methodological details are described with greater clarity and precision to ensure full reproducibility of the workflow.*

**3. Reproducibility:**

**Weaknesses: The role of generative AI (Claude AI) in manuscript preparation raises questions about authorship integrity and scientific interpretation.**

**Response:**

*We appreciate this important point and have addressed it directly in the revised manuscript. We added a formal Declaration of Generative AI Use, clarifying that Claude Sonnet 4.6 was used only to assist with literature synthesis, organization of manuscript sections, interpretation of visual outputs into narrative text, and improvement of writing clarity and terminology consistency. We explicitly state that all genomic analyses, statistical analyses, hypothesis generation, biological interpretation, and final scientific conclusions were performed and verified by the human authors, who take full responsibility for the content of the manuscript.*

**4. Contribution to the Field:**

**Weaknesses: Incremental contribution, overstated clinical implications, and omission of landmark studies limit novelty and relevance.**

**Response:**

*We appreciate this assessment and have revised the manuscript accordingly. We now position the work more appropriately as a large-scale descriptive and hypothesis-generating genomic survey of the S. maltophilia resistome. We have removed or softened statements that could be interpreted as implying immediate clinical application, particularly with respect to empiric therapy guidance or outbreak investigation. In addition, we updated the Introduction and Discussion to include relevant landmark studies, including work on L1/L2 β-lactamases and integrative conjugative elements, to better contextualize our findings within the established literature.*

**5. Sampling Biases and Missing Metadata:**

**Uneven geographic and temporal representation introduces potential confounding variables. Addressable through sensitivity analyses or alternative approaches (e.g., stratified analyses).**

**Response:**

*We agree. In response, we revised the temporal analysis to improve robustness by using weighted and unweighted regression approaches and by excluding years with fewer than two isolate reports. We also revised the Discussion to explicitly acknowledge that unequal sampling across countries, years, and metadata categories limits the epidemiological interpretation of observed patterns. Rather than making strong population-level claims, we now interpret these trends cautiously and emphasize that they may reflect both biological signals and biases in public genome deposition.*

**6. Lack of Experimental Validation:**

**Predicted AMR phenotypes are not experimentally validated, limiting translational relevance. Authors could acknowledge this limitation and discuss plans for future validation.**

**Response:**

*We agree fully and have revised the manuscript to emphasize this limitation. The Discussion now states clearly that our resistance inferences are based on genomic prediction rather than direct phenotypic validation, and therefore should not be interpreted as definitive evidence of expressed resistance in clinical settings. We also added a forward-looking statement indicating that future work should integrate matched phenotypic susceptibility testing, clinical metadata, and where possible functional validation of key resistance determinants.*

**7. Overstated Clinical Implications:**

**Claims about outbreak investigations and empiric therapy guidance exceed the scope of the data. Correctable by tempering language and focusing on exploratory findings.**

**Response:**

*We appreciate this comment and agree. We revised the manuscript throughout to avoid overstating the translational or clinical significance of our findings. Statements suggesting direct application to outbreak investigation or empiric therapy guidance have been removed or substantially moderated. The revised text now consistently presents the study as exploratory, intended to provide a genomic framework and generate hypotheses for future epidemiological and experimental work.*

**8. Ethical Concerns Related to Generative AI Use:**

**Role of Claude AI in manuscript preparation is unclear, raising questions about compliance with journal policies. Addressable by clarifying AI’s role and ensuring human oversight for all scientific interpretations.**

**Response:**

*We thank the Editor for raising this important issue. We have now included a clear Declaration of Generative AI Use in the manuscript. This statement specifies that Claude Sonnet 4.6 was used only for assistance with language improvement, literature synthesis support, organizational refinement, and transformation of analytical outputs into prose. We further clarify that all scientific decisions, data analyses, interpretations, and conclusions were performed and validated by the authors, in full compliance with authorship responsibilities.*

**9. Omission of Landmark Studies:**

**Missing citations (e.g., L1/L2 β-lactamase characterization, integrative conjugative elements) weaken context. Easily correctable by updating references.**

**Response:**

*We agree and have revised the manuscript to include additional landmark references relevant to S. maltophilia resistance biology. These include foundational and more recent work on L1/L2 β-lactamases, intrinsic resistance mechanisms, and integrative conjugative elements/mobile genetic determinants, thereby improving the biological context and relevance of our interpretation.*

**The authors should address the following key areas:**

**1. Perform sensitivity analyses to mitigate sampling biases and missing metadata.**

**Response:**

*Addressed. We implemented a more robust temporal analysis using weighted and unweighted regression and excluded years with fewer than two isolate reports. We also expanded the Discussion to explicitly address the implications of metadata incompleteness and unequal geographic/temporal sampling.*

**2. Acknowledge the exploratory nature of the study and avoid overstating clinical implications.**

**Response:**

*Addressed. We revised the Abstract, Discussion, and Conclusion to frame the study as exploratory and hypothesis-generating, and we removed or moderated statements suggesting direct clinical or outbreak-management application.*

**3. Provide raw data (e.g., accession numbers, scripts) and clarify the role of generative AI in manuscript preparation.**

**Response:**

*Addressed. We clarified the analytical workflow and dataset reporting in the revised manuscript and added a formal Declaration of Generative AI Use specifying the limited supportive role of Claude Sonnet 4.6 and the complete scientific oversight of the authors.*

**4. Include missing landmark studies to strengthen context and relevance.**

**Response:**

*Addressed. We incorporated additional key references, including studies on L1/L2 β-lactamases and integrative conjugative elements, to strengthen the contextual framework of the manuscript.*

**5. Discuss limitations and plans for future experimental validation.**

**Response:**

*Addressed. We expanded the limitations in the Discussion and explicitly noted the absence of phenotypic validation. We also outlined the need for future studies integrating phenotypic AST, clinical metadata, and functional experiments.*

**Reviewer #1**

**The manuscript presents an in silico analysis of 2,389 genomes annotated as Stenotrophomonas maltophilia retrieved from public databases, aiming to characterize antimicrobial resistance gene prevalence, temporal trends, and sequence type distribution. While the dataset size is large and the topic is relevant, the analytical framework and methodological rigor are insufficient to support the conclusions. Several fundamental issues related to species validation, genomic analysis depth, and interpretation substantially limit the reliability of the results.**

**Response:**

*We thank Reviewer #1 for the careful and critical assessment. We have revised the manuscript substantially to improve methodological clarity, strengthen interpretation, and better align our conclusions with the scope of the data. Importantly, species identity was confirmed using ANI, and this has now been explicitly clarified in the revised Methods. We also revised the temporal trend analysis using weighted and unweighted regression and excluded years with fewer than two isolate reports to reduce instability in sparsely represented periods. In addition, we moderated interpretations throughout the manuscript and expanded the limitations section to reflect the exploratory nature of the study.*

**Given these concerns, I do not believe the manuscript meets the methodological standards required for publication.**

**Response:**

*We respectfully appreciate the Reviewer’s concerns and have addressed them through major revision. We believe the revised version is methodologically stronger, more transparent, and more cautious in interpretation, particularly with respect to taxonomic confirmation, temporal analysis, limitations, and the scope of the conclusions.*

**Major Concerns**

**1. Lack of Species Confirmation**

**A major methodological concern is the absence of taxonomic validation of the genome dataset.**

**The authors retrieved genomes annotated as S. maltophilia from NCBI and applied quality filtering using CheckM completeness and contamination thresholds. However, no genomic confirmation of species identity was performed.**

**This is problematic because the Stenotrophomonas maltophilia complex contains multiple closely related species and cryptic lineages, and public genome repositories contain numerous misannotated assemblies. Without validation using approaches such as:**

**Average Nucleotide Identity (ANI) against the type strain**

**GTDB-based taxonomic classification**

**core genome phylogeny**

**the dataset may include genomes belonging to other Stenotrophomonas species. This could significantly bias downstream analyses of resistance gene prevalence, MLST distribution, and temporal trends. Therefore, the reliability of the dataset is uncertain.**

**Response:**

*We thank the Reviewer for highlighting this critical issue. Following this comment, we carefully re-evaluated the entire genome dataset and performed species confirmation using FastANI against the* Stenotrophomonas maltophilia *reference with a ≥95% ANI threshold for species-level assignment. This verification step revealed that a substantial number of genomes initially annotated as* S. maltophilia *in public repositories did not meet the required identity threshold. After applying this confirmation criterion, the final dataset was reduced to 1,239 genomes, with nearly half of the initially retrieved assemblies excluded due to insufficient ANI support for species assignment.*

*We appreciate the Reviewer’s suggestion, as this correction significantly improves the taxonomic accuracy and reliability of the dataset. The revised Methods section now explicitly describes the FastANI validation step and the applied threshold, ensuring that only confidently confirmed* S. maltophilia *genomes were retained for downstream analyses.*

**2. Overly Simplified Analytical Framework**

**The analytical approach used in the manuscript is relatively limited for a study claiming large-scale genomic epidemiology.**

**The study relies mainly on:**

**MLST typing**

**presence/absence of AMR genes**

**simple statistical correlations**

**However, modern bacterial comparative genomics typically includes:**

**core genome phylogenetic analysis**

**pan-genome reconstruction**

**analysis of mobile genetic elements**

**genomic context of resistance genes**

**lineage-specific resistome variation**

**Without these analyses, the manuscript provides only descriptive statistics rather than meaningful genomic insights.**

**Response:**

*We appreciate this comment and agree that our analytical framework is more descriptive than a full comparative genomics study. We have revised the manuscript to more accurately reflect this scope and no longer present it as a comprehensive genomic epidemiology analysis in the broadest sense. Instead, we now frame the study as a large-scale genomic survey of resistome prevalence, temporal patterns, and ST-associated variation. We also acknowledge in the Discussion that additional analyses such as core genome phylogeny, pan-genome reconstruction, mobile genetic element analysis, and genomic context characterization would provide deeper biological insight and represent important future directions.*

**3. Overinterpretation of Intrinsic Resistance Determinants**

**The manuscript emphasizes the high prevalence of efflux pump genes (e.g., smeF, emrA, emrB, emrC) and β-lactamases (blaL1, blaL2). However, these genes represent well-established intrinsic resistance mechanisms in S. maltophilia and are expected to occur in the majority of genomes.**

**Therefore, their high prevalence does not constitute novel findings and should not be interpreted as evidence of evolving resistance dynamics.**

**Response:**

*We agree with the Reviewer. In the revised manuscript, we have clarified that these genes represent the intrinsic resistance backbone of S. maltophilia and that their high prevalence is expected rather than novel in itself. We have revised the Discussion to avoid presenting these determinants as evidence of newly emerging resistance dynamics. Instead, we now use them to define the stable intrinsic resistome background against which the more variable accessory resistance patterns can be interpreted.*

**4. Questionable Temporal Trend Analysis**

**The authors attempt to infer temporal trends in resistance gene prevalence across a time span from 1900 to 2025. However, genome sampling from public repositories is highly uneven across time and geography.**

**Because the study does not account for sampling bias or normalize dataset representation across years, the reported temporal patterns may simply reflect database submission trends rather than true evolutionary changes.**

**Response:**

*We thank the Reviewer for this important point. In response, we revised the temporal analysis to improve robustness. Specifically, we used both weighted and unweighted regression models and removed years with fewer than two isolate reports to reduce instability caused by sparse sampling. We also revised the text to interpret temporal trends with much greater caution and explicitly acknowledge that patterns may reflect both true biological signal and public database sampling/submission bias. Thus, the revised manuscript no longer presents temporal trends as definitive evidence of evolutionary change.*

**5. Limited Biological Insight**

**Overall, the study mainly reports descriptive statistics of AMR gene frequency across publicly available genomes. The analysis does not sufficiently explore the biological context of resistance genes, such as:**

**association with mobile genetic elements**

**genomic neighborhood analysis**

**lineage-specific resistance patterns**

**evolutionary mechanisms underlying resistance dissemination**

**As a result, the study provides limited new biological insights.**

**Response:**

*We appreciate this thoughtful comment. We agree that the present study is primarily descriptive and that deeper biological context would strengthen mechanistic interpretation. In the revised manuscript, we have explicitly acknowledged this limitation and reframed the contribution of the work as establishing a broad genomic overview and a hypothesis-generating foundation for future studies. We also note that follow-up analyses focused on mobile genetic elements, genomic neighborhoods, and evolutionary dissemination mechanisms are important next steps beyond the current study design.*

**Minor Comments**

**Several grammatical and stylistic issues are present throughout the manuscript and require language editing.**

**Response:**

*We agree and have carefully revised the manuscript for grammar, clarity, readability, and consistency of scientific terminology. We also disclose that Claude Sonnet 4.6 was used only to improve writing quality, while all scientific content and interpretation remained under full author control.*

**The description of genome retrieval and filtering criteria should be clarified.**

**Response:**

*Addressed. We revised the Methods to clarify the genome retrieval process, quality filtering criteria, and the ANI-based species confirmation step used for final inclusion of isolates.*

**Figures require clearer legends describing clustering methods and color scales.**

**Response:**

*Addressed. We revised the figure legends to provide clearer descriptions of clustering approaches, annotation schemes, and color scales to improve interpretability.*

**The discussion section is lengthy and contains speculative interpretations that are not strongly supported by the results.**

**Response:**

*We agree and revised the Discussion substantially. The text has been streamlined, speculative statements have been removed or tempered, and the interpretation has been aligned more closely with the actual results and the acknowledged limitations of the dataset.*

**Although the dataset analyzed is large, the methodological framework and analytical depth are insufficient to support the conclusions drawn by the authors. In particular, the absence of species confirmation, limited genomic analyses, and potential sampling biases substantially weaken the study.**

**Response:**

*We thank the Reviewer for this important comment. In the revised manuscript, we substantially expanded the analytical framework to address these concerns.*

*First, species identity was confirmed using FastANI with a ≥95% ANI threshold against the Stenotrophomonas maltophilia reference genome. After validation, the dataset was reduced to 1,239 confirmed isolates, with nearly half of the initially retrieved genomes excluded due to insufficient species-level similarity. This correction significantly improves the taxonomic accuracy and reliability of the dataset (see Methods).*

*In addition, several new genomic analyses were incorporated to increase the analytical depth of the study. These include: (i) a resistome structure analysis based on resistance gene presence patterns identifying 73 distinct profiles across the dataset (Figure 9); (ii) resistance gene co‑occurrence network and accessory resistome architecture analyses across major sequence types (Figure 10); (iii) comparative analysis of resistance gene prevalence between clinical and non‑clinical isolates (Figure 7); and (iv) expanded temporal reconstruction of resistance gene, plasmid, and virulence determinant emergence (Figure 5).*

*Detailed descriptions of these analyses and their interpretation have been added to the Results and Discussion sections. These additions substantially strengthen the genomic scope and analytical depth of the manuscript.*
