# Supplementary material for: In silico antimicrobial resistance profiling of 1,240 Stenotrophomonas maltophilia genomes: AMR gene prevalence, temporal evolution, and ST-specific associations
Source: PLoS One. 2026 Jul 9;21(7):e0350669. doi: 10.1371/journal.pone.0350669 (PMC13349159; doi:10.1371/journal.pone.0350669)
Supplement: Peer review history

# Peer review: Decision Letter 0

**DOI:** 10.1371/journal.pone.0350669.r001

**Authors:** Abdelwahab Omri

1 Apr 2026

PONE-D-26-05406

In Silico Antimicrobial Resistance Profiling of 2,389 Stenotrophomonas maltophilia Genomes: AMR Gene Prevalence, Temporal Evolution, and ST-Specific Associations

PLOS One

Dear Dr. Sholeh,

Thank you for submitting your manuscript to PLOS ONE. After careful consideration, we feel that it has merit but does not fully meet PLOS ONE’s publication criteria as it currently stands. Therefore, we invite you to submit a revised version of the manuscript that addresses the points raised during the review process.

The manuscript presents a large-scale genomic analysis of antimicrobial resistance (AMR) in *Stenotrophomonas maltophilia* , leveraging 2,389 high-quality genomes to investigate AMR gene prevalence, temporal trends, and sequence type (ST)-specific resistance profiles. The study addresses an important gap in understanding the genomic epidemiology of this multidrug-resistant pathogen and contributes valuable insights into intrinsic resistance mechanisms, clonal expansion, and geographic variability.

Strengths include the robust dataset, stringent quality-control measures, and transparent reporting of methods. However, significant weaknesses—particularly the reliance on publicly available data, lack of experimental validation, overstated clinical implications, and ethical concerns related to generative AI use—undermine the manuscript’s overall impact. While the findings are biologically plausible and methodologically sound, they fall short of transformative contributions due to unresolved limitations.

**Scientific Rigor:**

- Weaknesses: Sampling biases, uneven metadata availability, and lack of experimental validation for predicted AMR phenotypes reduce confidence in findings.
**Methodological Transparency:**
  Weaknesses: Missing details on raw data availability (e.g., accession numbers, scripts) and parameters used for analyses hinder reproducibility.
- **Reproducibility:**
  Weaknesses: The role of generative AI (Claude AI) in manuscript preparation raises questions about authorship integrity and scientific interpretation.
- **Contribution to the Field:**
  Weaknesses: Incremental contribution, overstated clinical implications, and omission of landmark studies limit novelty and relevance.
  5.**Sampling Biases and Missing Metadata:**
  Uneven geographic and temporal representation introduces potential confounding variables. Addressable through sensitivity analyses or alternative approaches (e.g., stratified analyses).
- **Lack of Experimental Validation:**
  Predicted AMR phenotypes are not experimentally validated, limiting translational relevance. Authors could acknowledge this limitation and discuss plans for future validation.

**Overstated Clinical Implications:**

- Claims about outbreak investigations and empiric therapy guidance exceed the scope of the data. Correctable by tempering language and focusing on exploratory findings.
- **Ethical Concerns Related to Generative AI Use:**
  Role of Claude AI in manuscript preparation is unclear, raising questions about compliance with journal policies. Addressable by clarifying AI’s role and ensuring human oversight for all scientific interpretations.

**Omission of Landmark Studies:**

- Missing citations (e.g., L1/L2 β-lactamase characterization, integrative conjugative elements) weaken context. Easily correctable by updating references.

Based on the above evaluation, the manuscript does not currently meet PLOS ONE’s threshold criteria for publication. While the study demonstrates scientific rigor and methodological transparency, the weaknesses-particularly sampling biases, lack of experimental validation, and overstated clinical implications-significantly detract from its impact. These issues are correctable, however, and the manuscript has potential to make a meaningful contribution after major revisions.

The authors should address the following key areas:

1. Perform sensitivity analyses to mitigate sampling biases and missing metadata.
2. Acknowledge the exploratory nature of the study and avoid overstating clinical implications.
3. Provide raw data (e.g., accession numbers, scripts) and clarify the role of generative AI in manuscript preparation.
4. Include missing landmark studies to strengthen context and relevance.
5. Discuss limitations and plans for future experimental validation.

Please submit your revised manuscript by May 16 2026 11:59PM. If you will need more time than this to complete your revisions, please reply to this message or contact the journal office at plosone@plos.org. When you're ready to submit your revision, log on to https://www.editorialmanager.com/pone/ and select the 'Submissions Needing Revision' folder to locate your manuscript file.

Please include the following items when submitting your revised manuscript:

- A letter that responds to each point raised by the academic editor and reviewer(s). You should upload this letter as a separate file labeled 'Response to Reviewers'.
- A marked-up copy of your manuscript that highlights changes made to the original version. You should upload this as a separate file labeled 'Revised Manuscript with Track Changes'.
- An unmarked version of your revised paper without tracked changes. You should upload this as a separate file labeled 'Manuscript'.

If you would like to make changes to your financial disclosure, please include your updated statement in your cover letter. Guidelines for resubmitting your figure files are available below the reviewer comments at the end of this letter.

If applicable, we recommend that you deposit your laboratory protocols in protocols.io to enhance the reproducibility of your results. Protocols.io assigns your protocol its own identifier (DOI) so that it can be cited independently in the future. For instructions see: https://journals.plos.org/plosone/s/submission-guidelines#loc-laboratory-protocols. Additionally, PLOS ONE offers an option for publishing peer-reviewed Lab Protocol articles, which describe protocols hosted on protocols.io. Read more information on sharing protocols at https://plos.org/protocols?utm_medium=editorial-email&utm_source=authorletters&utm_campaign=protocols.

As the corresponding author, your ORCID iD is verified in the submission system and will appear in the published article. PLOS supports the use of ORCID, and we encourage all coauthors to register for an ORCID iD and use it as well. Please encourage your coauthors to verify their ORCID iD within the submission system before final acceptance, as unverified ORCID iDs will not appear in the published article. *Only*  the individual author can complete the verification step; PLOS staff *cannot*  verify ORCID iDs on behalf of authors.

We look forward to receiving your revised manuscript.

Kind regards,

Abdelwahab Omri, Pharm B, Ph.D

Academic Editor

PLOS One

Journal Requirements:

When submitting your revision, we need you to address these additional requirements.

1. Please ensure that your manuscript meets PLOS ONE's style requirements, including those for file naming. The PLOS ONE style templates can be found at

https://journals.plos.org/plosone/s/file?id=wjVg/PLOSOne_formatting_sample_main_body.pdf and

https://journals.plos.org/plosone/s/file?id=ba62/PLOSOne_formatting_sample_title_authors_affiliations.pdf

2. In your Methods section, please provide additional information regarding the permits you obtained for the work. Please ensure you have included the full name of the authority that approved the field site access and, if no permits were required, a brief statement explaining why.

3. Thank you for stating the following in your Competing Interests section:

"NO authors have competing interests"

Please complete your Competing Interests on the online submission form to state any Competing Interests. If you have no competing interests, please state "The authors have declared that no competing interests exist.", as detailed online in our guide for authors at http://journals.plos.org/plosone/s/submit-now

This information should be included in your cover letter; we will change the online submission form on your behalf.

4. Please include captions for your Supporting Information files at the end of your manuscript, and update any in-text citations to match accordingly. Please see our Supporting Information guidelines for more information: http://journals.plos.org/plosone/s/supporting-information.

5. If the reviewer comments include a recommendation to cite specific previously published works, please review and evaluate these publications to determine whether they are relevant and should be cited. There is no requirement to cite these works unless the editor has indicated otherwise.

Additional Editor Comments:

The manuscript presents a large-scale genomic analysis of antimicrobial resistance (AMR) in Stenotrophomonas maltophilia, leveraging 2,389 high-quality genomes to investigate AMR gene prevalence, temporal trends, and sequence type (ST)-specific resistance profiles. The study addresses an important gap in understanding the genomic epidemiology of this multidrug-resistant pathogen and contributes valuable insights into intrinsic resistance mechanisms, clonal expansion, and geographic variability.

Strengths include the robust dataset, stringent quality-control measures, and transparent reporting of methods. However, significant weaknesses—particularly the reliance on publicly available data, lack of experimental validation, overstated clinical implications, and ethical concerns related to generative AI use—undermine the manuscript’s overall impact. While the findings are biologically plausible and methodologically sound, they fall short of transformative contributions due to unresolved limitations.

1. Scientific Rigor:

o Weaknesses: Sampling biases, uneven metadata availability, and lack of experimental validation for predicted AMR phenotypes reduce confidence in findings.

2. Methodological Transparency:

o Weaknesses: Missing details on raw data availability (e.g., accession numbers, scripts) and parameters used for analyses hinder reproducibility.

3. Reproducibility:

o Weaknesses: The role of generative AI (Claude AI) in manuscript preparation raises questions about authorship integrity and scientific interpretation.

4. Contribution to the Field:

o Weaknesses: Incremental contribution, overstated clinical implications, and omission of landmark studies limit novelty and relevance.

5. Sampling Biases and Missing Metadata:

o Uneven geographic and temporal representation introduces potential confounding variables. Addressable through sensitivity analyses or alternative approaches (e.g., stratified analyses).

6. Lack of Experimental Validation:

o Predicted AMR phenotypes are not experimentally validated, limiting translational relevance. Authors could acknowledge this limitation and discuss plans for future validation.

7. Overstated Clinical Implications:

o Claims about outbreak investigations and empiric therapy guidance exceed the scope of the data. Correctable by tempering language and focusing on exploratory findings.

8. Ethical Concerns Related to Generative AI Use:

o Role of Claude AI in manuscript preparation is unclear, raising questions about compliance with journal policies. Addressable by clarifying AI’s role and ensuring human oversight for all scientific interpretations.

9. Omission of Landmark Studies:

o Missing citations (e.g., L1/L2 β-lactamase characterization, integrative conjugative elements) weaken context. Easily correctable by updating references.

Based on the above evaluation, the manuscript does not currently meet PLOS ONE’s threshold criteria for publication. While the study demonstrates scientific rigor and methodological transparency, the weaknesses-particularly sampling biases, lack of experimental validation, and overstated clinical implications-significantly detract from its impact. These issues are correctable, however, and the manuscript has potential to make a meaningful contribution after major revisions.

The authors should address the following key areas:

1. Perform sensitivity analyses to mitigate sampling biases and missing metadata.

2. Acknowledge the exploratory nature of the study and avoid overstating clinical implications.

3. Provide raw data (e.g., accession numbers, scripts) and clarify the role of generative AI in manuscript preparation.

4. Include missing landmark studies to strengthen context and relevance.

5. Discuss limitations and plans for future experimental validation.

[Note: HTML markup is below. Please do not edit.]

Reviewers' comments:

Reviewer's Responses to Questions

**Comments to the Author**

1. Is the manuscript technically sound, and do the data support the conclusions?

The manuscript must describe a technically sound piece of scientific research with data that supports the conclusions. Experiments must have been conducted rigorously, with appropriate controls, replication, and sample sizes. The conclusions must be drawn appropriately based on the data presented.

Reviewer #1: Partly

**********

2. Has the statistical analysis been performed appropriately and rigorously?

Reviewer #1: Yes

**********

3. Have the authors made all data underlying the findings in their manuscript fully available?

The PLOS Data policy requires authors to make all data underlying the findings described in their manuscript fully available without restriction, with rare exception (please refer to the Data Availability Statement in the manuscript PDF file). The data should be provided as part of the manuscript or its supporting information, or deposited to a public repository. For example, in addition to summary statistics, the data points behind means, medians and variance measures should be available. If there are restrictions on publicly sharing data—e.g. participant privacy or use of data from a third party—those must be specified.

Reviewer #1: Yes

**********

4. Is the manuscript presented in an intelligible fashion and written in standard English?

PLOS ONE does not copyedit accepted manuscripts, so the language in submitted articles must be clear, correct, and unambiguous. Any typographical or grammatical errors should be corrected at revision, so please note any specific errors here.

Reviewer #1: No

**********

5. Review Comments to the Author

Please use the space provided to explain your answers to the questions above. You may also include additional comments for the author, including concerns about dual publication, research ethics, or publication ethics. (Please upload your review as an attachment if it exceeds 20,000 characters)

Reviewer #1: The manuscript presents an in silico analysis of 2,389 genomes annotated as Stenotrophomonas maltophilia retrieved from public databases, aiming to characterize antimicrobial resistance gene prevalence, temporal trends, and sequence type distribution. While the dataset size is large and the topic is relevant, the analytical framework and methodological rigor are insufficient to support the conclusions. Several fundamental issues related to species validation, genomic analysis depth, and interpretation substantially limit the reliability of the results.

Given these concerns, I do not believe the manuscript meets the methodological standards required for publication.

Major Concerns

1. Lack of Species Confirmation

A major methodological concern is the absence of taxonomic validation of the genome dataset.

The authors retrieved genomes annotated as S. maltophilia from NCBI and applied quality filtering using CheckM completeness and contamination thresholds. However, no genomic confirmation of species identity was performed.

This is problematic because the Stenotrophomonas maltophilia complex contains multiple closely related species and cryptic lineages, and public genome repositories contain numerous misannotated assemblies. Without validation using approaches such as:

Average Nucleotide Identity (ANI) against the type strain

GTDB-based taxonomic classification

core genome phylogeny

the dataset may include genomes belonging to other Stenotrophomonas species. This could significantly bias downstream analyses of resistance gene prevalence, MLST distribution, and temporal trends. Therefore, the reliability of the dataset is uncertain.

2. Overly Simplified Analytical Framework

The analytical approach used in the manuscript is relatively limited for a study claiming large-scale genomic epidemiology.

The study relies mainly on:

MLST typing

presence/absence of AMR genes

simple statistical correlations

However, modern bacterial comparative genomics typically includes:

core genome phylogenetic analysis

pan-genome reconstruction

analysis of mobile genetic elements

genomic context of resistance genes

lineage-specific resistome variation

Without these analyses, the manuscript provides only descriptive statistics rather than meaningful genomic insights.

3. Overinterpretation of Intrinsic Resistance Determinants

The manuscript emphasizes the high prevalence of efflux pump genes (e.g., smeF, emrA, emrB, emrC) and β-lactamases (blaL1, blaL2). However, these genes represent well-established intrinsic resistance mechanisms in S. maltophilia and are expected to occur in the majority of genomes.

Therefore, their high prevalence does not constitute novel findings and should not be interpreted as evidence of evolving resistance dynamics.

4. Questionable Temporal Trend Analysis

The authors attempt to infer temporal trends in resistance gene prevalence across a time span from 1900 to 2025. However, genome sampling from public repositories is highly uneven across time and geography.

Because the study does not account for sampling bias or normalize dataset representation across years, the reported temporal patterns may simply reflect database submission trends rather than true evolutionary changes.

5. Limited Biological Insight

Overall, the study mainly reports descriptive statistics of AMR gene frequency across publicly available genomes. The analysis does not sufficiently explore the biological context of resistance genes, such as:

association with mobile genetic elements

genomic neighborhood analysis

lineage-specific resistance patterns

evolutionary mechanisms underlying resistance dissemination

As a result, the study provides limited new biological insights.

Minor Comments

Several grammatical and stylistic issues are present throughout the manuscript and require language editing.

The description of genome retrieval and filtering criteria should be clarified.

Figures require clearer legends describing clustering methods and color scales.

The discussion section is lengthy and contains speculative interpretations that are not strongly supported by the results.

Although the dataset analyzed is large, the methodological framework and analytical depth are insufficient to support the conclusions drawn by the authors. In particular, the absence of species confirmation, limited genomic analyses, and potential sampling biases substantially weaken the study.

For these reasons, I recommend rejection of the manuscript.

**********

6. PLOS authors have the option to publish the peer review history of their article (what does this mean?). If published, this will include your full peer review and any attached files.

If you choose “no”, your identity will remain anonymous but your review may still be made public.

**Do you want your identity to be public for this peer review?**  For information about this choice, including consent withdrawal, please see our Privacy Policy.

Reviewer #1: No

**********

[NOTE: If reviewer comments were submitted as an attachment file, they will be attached to this email and accessible via the submission site. Please log into your account, locate the manuscript record, and check for the action link "View Attachments". If this link does not appear, there are no attachment files.]

To ensure your figures meet our technical requirements, please review our figure guidelines: https://journals.plos.org/plosone/s/figures

You may also use PLOS’s free figure tool, NAAS, to help you prepare publication quality figures: https://journals.plos.org/plosone/s/figures#loc-tools-for-figure-preparation.

NAAS will assess whether your figures meet our technical requirements by comparing each figure against our figure specifications.

# Author comment: Author response to Decision Letter 1

**DOI:** 10.1371/journal.pone.0350669.r002

14 May 2026

Response to Editor and Reviewer

We thank the Academic Editor and Reviewer #1 for their careful evaluation of our manuscript and for the constructive comments. We have revised the manuscript extensively to address all concerns. In particular, we clarified that Average Nucleotide Identity (ANI) was used for confirmation of included isolates, we strengthened the temporal regression analysis by applying weighted and unweighted regression approaches and excluding years with fewer than two isolate reports, we expanded the limitations in the Discussion, we tempered statements regarding clinical implications, we added clarification regarding generative AI use, and we updated the manuscript context with relevant landmark studies.

Additional Editor Comments:

The manuscript presents a large-scale genomic analysis of antimicrobial resistance (AMR) in Stenotrophomonas maltophilia, leveraging 2,389 high-quality genomes to investigate AMR gene prevalence, temporal trends, and sequence type (ST)-specific resistance profiles. The study addresses an important gap in understanding the genomic epidemiology of this multidrug-resistant pathogen and contributes valuable insights into intrinsic resistance mechanisms, clonal expansion, and geographic variability. Strengths include the robust dataset, stringent quality-control measures, and transparent reporting of methods. However, significant weaknesses—particularly the reliance on publicly available data, lack of experimental validation, overstated clinical implications, and ethical concerns related to generative AI use—undermine the manuscript’s overall impact. While the findings are biologically plausible and methodologically sound, they fall short of transformative contributions due to unresolved limitations.

Response:

We thank the Editor for this balanced assessment. We have revised the manuscript to better align the scope of our conclusions with the nature of the dataset and the analytical design. Specifically, we now more clearly present the study as a large-scale exploratory genomic analysis rather than a transformative or directly practice-changing investigation. We have also strengthened the manuscript by clarifying species confirmation using ANI, expanding the Discussion of sampling bias and metadata limitations, explicitly acknowledging the lack of experimental validation, tempering clinical interpretation, and adding a transparent Declaration of Generative AI Use with clear author oversight.

1. Scientific Rigor:

Weaknesses: Sampling biases, uneven metadata availability, and lack of experimental validation for predicted AMR phenotypes reduce confidence in findings.

Response:

We agree and have addressed these issues in the revised manuscript. We now explicitly acknowledge that the use of publicly available genomes introduces geographic, temporal, and source-related sampling biases, as well as uneven metadata completeness. To improve robustness of the temporal analysis, we used both weighted and unweighted regression models and excluded years with fewer than two isolate reports, thereby reducing distortion from sparsely represented years. In addition, we expanded the Discussion to state clearly that predicted AMR profiles are based on in silico gene detection and do not substitute for phenotypic susceptibility testing. We now frame these results as hypothesis-generating and suitable for future validation rather than definitive evidence of expressed resistance phenotypes.

2. Methodological Transparency:

Weaknesses: Missing details on raw data availability (e.g., accession numbers, scripts) and parameters used for analyses hinder reproducibility.

Response:

We thank the Editor for this comment. We have revised the manuscript to ensure that all accession numbers and related identifiers are fully provided in the Supplementary File, and that methodological details are described with greater clarity and precision to ensure full reproducibility of the workflow.

3. Reproducibility:

Weaknesses: The role of generative AI (Claude AI) in manuscript preparation raises questions about authorship integrity and scientific interpretation.

Response:

We appreciate this important point and have addressed it directly in the revised manuscript. We added a formal Declaration of Generative AI Use, clarifying that Claude Sonnet 4.6 was used only to assist with literature synthesis, organization of manuscript sections, interpretation of visual outputs into narrative text, and improvement of writing clarity and terminology consistency. We explicitly state that all genomic analyses, statistical analyses, hypothesis generation, biological interpretation, and final scientific conclusions were performed and verified by the human authors, who take full responsibility for the content of the manuscript.

________________________________________

4. Contribution to the Field:

Weaknesses: Incremental contribution, overstated clinical implications, and omission of landmark studies limit novelty and relevance.

Response:

We appreciate this assessment and have revised the manuscript accordingly. We now position the work more appropriately as a large-scale descriptive and hypothesis-generating genomic survey of the S. maltophilia resistome. We have removed or softened statements that could be interpreted as implying immediate clinical application, particularly with respect to empiric therapy guidance or outbreak investigation. In addition, we updated the Introduction and Discussion to include relevant landmark studies, including work on L1/L2 β-lactamases and integrative conjugative elements, to better contextualize our findings within the established literature.

5. Sampling Biases and Missing Metadata:

Uneven geographic and temporal representation introduces potential confounding variables. Addressable through sensitivity analyses or alternative approaches (e.g., stratified analyses).

Response:

We agree. In response, we revised the temporal analysis to improve robustness by using weighted and unweighted regression approaches and by excluding years with fewer than two isolate reports. We also revised the Discussion to explicitly acknowledge that unequal sampling across countries, years, and metadata categories limits the epidemiological interpretation of observed patterns. Rather than making strong population-level claims, we now interpret these trends cautiously and emphasize that they may reflect both biological signals and biases in public genome deposition.

6. Lack of Experimental Validation:

Predicted AMR phenotypes are not experimentally validated, limiting translational relevance. Authors could acknowledge this limitation and discuss plans for future validation.

Response:

We agree fully and have revised the manuscript to emphasize this limitation. The Discussion now states clearly that our resistance inferences are based on genomic prediction rather than direct phenotypic validation, and therefore should not be interpreted as definitive evidence of expressed resistance in clinical settings. We also added a forward-looking statement indicating that future work should integrate matched phenotypic susceptibility testing, clinical metadata, and where possible functional validation of key resistance determinants.

7. Overstated Clinical Implications:

Claims about outbreak investigations and empiric therapy guidance exceed the scope of the data. Correctable by tempering language and focusing on exploratory findings.

Response:

We appreciate this comment and agree. We revised the manuscript throughout to avoid overstating the translational or clinical significance of our findings. Statements suggesting direct application to outbreak investigation or empiric therapy guidance have been removed or substantially moderated. The revised text now consistently presents the study as exploratory, intended to provide a genomic framework and generate hypotheses for future epidemiological and experimental work.

8. Ethical Concerns Related to Generative AI Use:

Role of Claude AI in manuscript preparation is unclear, raising questions about compliance with journal policies. Addressable by clarifying AI’s role and ensuring human oversight for all scientific interpretations.

Response:

We thank the Editor for raising this important issue. We have now included a clear Declaration of Generative AI Use in the manuscript. This statement specifies that Claude Sonnet 4.6 was used only for assistance with language improvement, literature synthesis support, organizational refinement, and transformation of analytical outputs into prose. We further clarify that all scientific decisions, data analyses, interpretations, and conclusions were performed and validated by the authors, in full compliance with authorship responsibilities.

9. Omission of Landmark Studies:

Missing citations (e.g., L1/L2 β-lactamase characterization, integrative conjugative elements) weaken context. Easily correctable by updating references.

Response:

We agree and have revised the manuscript to include additional landmark references relevant to S. maltophilia resistance biology. These include foundational and more recent work on L1/L2 β-lactamases, intrinsic resistance mechanisms, and integrative conjugative elements/mobile genetic determinants, thereby improving the biological context and relevance of our interpretation.

The authors should address the following key areas:

1. Perform sensitivity analyses to mitigate sampling biases and missing metadata.

Response:

Addressed. We implemented a more robust temporal analysis using weighted and unweighted regression and excluded years with fewer than two isolate reports. We also expanded the Discussion to explicitly address the implications of metadata incompleteness and unequal geographic/temporal sampling.

2. Acknowledge the exploratory nature of the study and avoid overstating clinical implications.

Response:

Addressed. We revised the Abstract, Discussion, and Conclusion to frame the study as exploratory and hypothesis-generating, and we removed or moderated statements suggesting direct clinical or outbreak-management application.

3. Provide raw data (e.g., accession numbers, scripts) and clarify the role of generative AI in manuscript preparation.

Response:

Addressed. We clarified the analytical workflow and dataset reporting in the revised manuscript and added a formal Declaration of Generative AI Use specifying the limited supportive role of Claude Sonnet 4.6 and the complete scientific oversight of the authors.

4. Include missing landmark studies to strengthen context and relevance.

Response:

Addressed. We incorporated additional key references, including studies on L1/L2 β-lactamases and integrative conjugative elements, to strengthen the contextual framework of the manuscript.

5. Discuss limitations and plans for future experimental validation.

Response:

Addressed. We expanded the limitations in the Discussion and explicitly noted the absence of phenotypic validation. We also outlined the need for future studies integrating phenotypic AST, clinical metadata, and functional experiments.

Reviewer #1

The manuscript presents an in silico analysis of 2,389 genomes annotated as Stenotrophomonas maltophilia retrieved from public databases, aiming to characterize antimicrobial resistance gene prevalence, temporal trends, and sequence type distribution. While the dataset size is large and the topic is relevant, the analytical framework and methodological rigor are insufficient to support the conclusions. Several fundamental issues related to species validation, genomic analysis depth, and interpretation substantially limit the reliability of the results.

Response:

We thank Reviewer #1 for the careful and critical assessment. We have revised the manuscript substantially to improve methodological clarity, strengthen interpretation, and better align our conclusions with the scope of the data. Importantly, species identity was confirmed using ANI, and this has now been explicitly clarified in the revised Methods. We also revised the temporal trend analysis using weighted and unweighted regression and excluded years with fewer than two isolate reports to reduce instability in sparsely represented periods. In addition, we moderated interpretations throughout the manuscript and expanded the limitations section to reflect the exploratory nature of the study.

Given these concerns, I do not believe the manuscript meets the methodological standards required for publication.

Response:

We respectfully appreciate the Reviewer’s concerns and have addressed them through major revision. We believe the revised version is methodologically stronger, more transparent, and more cautious in interpretation, particularly with respect to taxonomic confirmation, temporal analysis, limitations, and the scope of the conclusions.

Major Concerns

1. Lack of Species Confirmation

A major methodological concern is the absence of taxonomic validation of the genome dataset.

The authors retrieved genomes annotated as S. maltophilia from NCBI and applied quality filtering using CheckM completeness and contamination thresholds. However, no genomic confirmation of species identity was performed.

This is problematic because the Stenotrophomonas maltophilia complex contains multiple closely related species and cryptic lineages, and public genome repositories contain numerous misannotated assemblies. Without validation using approaches such as:

Average Nucleotide Identity (ANI) against the type strain

GTDB-based taxonomic classification

core genome phylogeny

the dataset may include genomes belonging to other Stenotrophomonas species. This could significantly bias downstream analyses of resistance gene prevalence, MLST distribution, and temporal trends. Therefore, the reliability of the dataset is uncertain.

Response:

We thank the Reviewer for highlighting this critical issue. Following this comment, we carefully re-evaluated the entire genome dataset and performed species confirmation using FastANI against the Stenotrophomonas maltophilia reference with a ≥95% ANI threshold for species-level assignment. This verification step revealed that a substantial number of genomes initially annotated as S. maltophilia in public repositories did not meet the required identity threshold. After applying this confirmation criterion, the final dataset was reduced to 1,239 genomes, with nearly half of the initially retrieved assemblies excluded due to insufficient ANI support for species assignment.

We appreciate the Reviewer’s suggestion, as this correction significantly improves the taxonomic accuracy and reliability of the dataset. The revised Methods section now explicitly describes the FastANI validation step and the applied threshold, ensuring that only confidently confirmed S. maltophilia genomes were retained for downstream analyses.

2. Overly Simplified Analytical Framework

The analytical approach used in the manuscript is relatively limited for a study claiming large-scale genomic epidemiology.

The study relies mainly on:

MLST typing

presence/absence of AMR genes

simple statistical correlations

However, modern bacterial comparative genomics typically includes:

core genome phylogenetic analysis

pan-genome reconstruction

analysis of mobile genetic elements

genomic context of resistance genes

lineage-specific resistome variation

Without these analyses, the manuscript provides only descriptive statistics rather than meaningful genomic insights.

Response:

We appreciate this comment and agree that our analytical framework is more descriptive than a full comparative genomics study. We have revised the manuscript to more accurately reflect this scope and no longer present it as a comprehensive genomic epidemiology analysis in the broadest sense. Instead, we now frame the study as a large-scale genomic survey of resistome prevalence, temporal patterns, and ST-associated variation. We also acknowledge in the Discussion that additional analyses such as core genome phylogeny, pan-genome reconstruction, mobile genetic element analysis, and genomic context characterization would provide deeper biological insight and represent important future directions.

3. Overinterpretation of Intrinsic Resistance Determinants

The manuscript emphasizes the

Attachment Submitted filename: Response to Reviewers.docx

# Editor report: Decision Letter 1

**DOI:** 10.1371/journal.pone.0350669.r003

**Authors:** Abdelwahab Omri, Abdelwahab Omri

17 May 2026

In Silico Antimicrobial Resistance Profiling of 1,240 Stenotrophomonas maltophilia Genomes: AMR Gene Prevalence, Temporal Evolution, and ST-Specific Associations

PONE-D-26-05406R1

Dear Dr. Mohammad Sholeh,

We’re pleased to inform you that your manuscript has been judged scientifically suitable for publication and will be formally accepted for publication once it meets all outstanding technical requirements.

Within one week, you’ll receive an e-mail detailing the required amendments. When these have been addressed, you’ll receive a formal acceptance letter and your manuscript will be scheduled for publication.

An invoice will be generated when your article is formally accepted. Please note, if your institution has a publishing partnership with PLOS and your article meets the relevant criteria, all or part of your publication costs will be covered. Please make sure your user information is up-to-date by logging into Editorial Manager at Editorial Manager® and clicking the ‘Update My Information' link at the top of the page. For questions related to billing, please contact billing support.

If your institution or institutions have a press office, please notify them about your upcoming paper to help maximize its impact. If they’ll be preparing press materials, please inform our press team as soon as possible -- no later than 48 hours after receiving the formal acceptance. Your manuscript will remain under strict press embargo until 2 pm Eastern Time on the date of publication. For more information, please contact onepress@plos.org.

Kind regards,

Abdelwahab Omri, Pharm B, Ph.D, Laurentian University, Canada

Academic Editor

PLOS One

Additional Editor Comments (optional):

Accept

# Editor report: Acceptance letter

**DOI:** 10.1371/journal.pone.0350669.r004

**Authors:** Abdelwahab Omri, Abdelwahab Omri

PONE-D-26-05406R1

PLOS One

Dear Dr. Sholeh,

I'm pleased to inform you that your manuscript has been deemed suitable for publication in PLOS One. Congratulations! Your manuscript is now being handed over to our production team.

At this stage, our production department will prepare your paper for publication. This includes ensuring the following:

* All references, tables, and figures are properly cited

* All relevant supporting information is included in the manuscript submission,

* There are no issues that prevent the paper from being properly typeset

You will receive further instructions from the production team, including instructions on how to review your proof when it is ready. Please keep in mind that we are working through a large volume of accepted articles, so please give us a few days to review your paper and let you know the next and final steps.

Lastly, if your institution or institutions have a press office, please let them know about your upcoming paper now to help maximize its impact. If they'll be preparing press materials, please inform our press team within the next 48 hours. Your manuscript will remain under strict press embargo until 2 pm Eastern Time on the date of publication. For more information, please contact onepress@plos.org.

You will receive an invoice from PLOS for your publication fee after your manuscript has reached the completed accept phase. If you receive an email requesting payment before acceptance or for any other service, this may be a phishing scheme. Learn how to identify phishing emails and protect your accounts at https://explore.plos.org/phishing.

If we can help with anything else, please email us at customercare@plos.org.

Thank you for submitting your work to PLOS ONE and supporting open access.

Kind regards,

PLOS ONE Editorial Office Staff

on behalf of

Dr. Abdelwahab Omri

Academic Editor

PLOS One
